# Components of Coated Vesicles and Nuclear Pore Complexes Share a Common Molecular Architecture

**DOI:** 10.1371/journal.pbio.0020380

**Published:** 2004-11-02

**Authors:** Damien Devos, Svetlana Dokudovskaya, Frank Alber, Rosemary Williams, Brian T Chait, Andrej Sali, Michael P Rout

**Affiliations:** **1**Departments of Biopharmaceutical Sciences and Pharmaceutical Chemistry and California Institute for Quantitative Biomedical Research, University of CaliforniaSan Francisco, CaliforniaUnited States of America; **2**Laboratory of Cellular and Structural Biology, Rockefeller UniversityNew York, New YorkUnited States of America; **3**Laboratory of Mass Spectrometry and Gaseous Ion Chemistry, Rockefeller UniversityNew York, New YorkUnited States of America

## Abstract

Numerous features distinguish prokaryotes from eukaryotes, chief among which are the distinctive internal membrane systems of eukaryotic cells. These membrane systems form elaborate compartments and vesicular trafficking pathways, and sequester the chromatin within the nuclear envelope. The nuclear pore complex is the portal that specifically mediates macromolecular trafficking across the nuclear envelope. Although it is generally understood that these internal membrane systems evolved from specialized invaginations of the prokaryotic plasma membrane, it is not clear how the nuclear pore complex could have evolved from organisms with no analogous transport system. Here we use computational and biochemical methods to perform a structural analysis of the seven proteins comprising the yNup84/vNup107–160 subcomplex, a core building block of the nuclear pore complex. Our analysis indicates that all seven proteins contain either a β-propeller fold, an α-solenoid fold, or a distinctive arrangement of both, revealing close similarities between the structures comprising the yNup84/vNup107–160 subcomplex and those comprising the major types of vesicle coating complexes that maintain vesicular trafficking pathways. These similarities suggest a common evolutionary origin for nuclear pore complexes and coated vesicles in an early membrane-curving module that led to the formation of the internal membrane systems in modern eukaryotes.

## Abstract 2

Structural similarities between the proteins of a nuclear pore subcomplex and proteins comprising vesicle coating complexes indicate a common origin for nuclear pore complexes and coated vesicles

## Introduction

The ability to sharply curve membranes was a defining event in the evolution of early eukaryotes, allowing the formation of endomembrane systems (Blobel 1980). In modern eukaryotes, these systems have become elaborate internal membranes, such as the Golgi apparatus, the endoplasmic reticulum (ER), and the nuclear envelope (NE). To date three major kinds of transport vesicles, distinguished by the compositions of their protein coat complexes, have been shown to traffic between these internal membranes and the plasma membrane: First, the clathrin/adaptin complexes are responsible for endocytosis and vesicular trafficking between the Golgi, lysosomes, and endosomes; second, the COPI complex mediates intra-Golgi and Golgi-to-ER trafficking; and lastly, the COPII complex supports vesicle movement from the ER to the Golgi (reviewed in Kirchhausen 2000a, 2000b; Boehm and Bonifacino 2001; Bonifacino and Lippincott-Schwartz 2003; Lippincott-Schwartz and Liu 2003).

The NE is contiguous with the ER and delineates the nucleus. It is made of an inner and outer membrane that together form a barrier between the nucleoplasm and the cytoplasm. This barrier is perforated by nuclear pore complexes (NPCs), which form pores between the inner and outer NE membranes by stabilizing a sharply curved section of connecting pore membrane. NPCs are approximately 50-MDa octagonally symmetric cylinders that function as the only known mediators of nucleocytoplasmic exchange; while permitting the free flow of small molecules, they restrict macromolecular trafficking to selected cargoes that are recognized by cognate transport factors. NPCs are found in all eukaryotic cells and are composed of a broadly conserved set of proteins, termed nups (reviewed in Rout and Aitchison 2001; Bednenko et al. 2003; Rout et al. 2003; Suntharalingam and Wente 2003; Fahrenkrog et al. 2004). Although the nups have been fully cataloged for both yeast *(Saccharomyces)* (Rout et al. 2000) and vertebrates (Cronshaw et al. 2002), there is currently little information concerning their origin and evolution. To this end, protein structures are helpful because it is easier to recognize similarities in structure than in sequence, especially for distantly related proteins. Thus, we have characterized the structures of seven proteins forming a core building block of the NPC, termed the yNup84 subcomplex in *Saccharomyces* and the vNup107–160 subcomplex in vertebrates. These structures reveal how the nuclear pore complex could have evolved from organisms with no analogous transport system.

## Results

**Figure 1:**
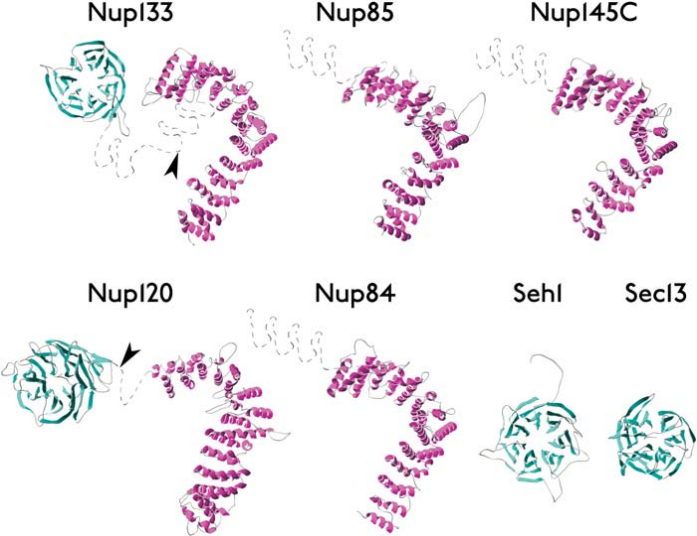
Ribbon Representation of Nup Models β-sheets (β-propellers) are colored cyan and α-helices (α-solenoids) are colored magenta. Gray dashed lines indicate regions that were not modeled. Arrowheads indicate the positions of high proteolytic susceptibility (see Figures 2 and 3).

The yNup84/vNup107–160 subcomplex has a molecular weight of approximately 600 kDa and has been shown in yeast to be flexible (Siniossoglou et al. 1996; Siniossoglou et al. 2000; Lutzmann et al. 2002), presenting a considerable challenge to conventional experimental methods for structure determination; thus, we used a computational approach that relies on a database of experimentally determined structures (Marti-Renom et al. 2000). We first focused on the component nups of the yNup84 subcomplex: ySeh1, ySec13, yNup84, yNup85, yNup120, yNup133, and yNup145C, whose corresponding vertebrate homologs are, respectively, vSec13 l, vSec13R, vNup107, vNup75, vNup160, vNup133, and vNup96 (Siniossoglou et al. 1996; Fontoura et al. 1999; Siniossoglou et al. 2000; Cronshaw et al. 2002; Lutzmann et al. 2002; Boehmer et al. 2003; Harel et al. 2003; Walther et al. 2003; Loiodice et al. 2004). For putative domains in each of these nups, we first applied two threading programs to assign structure folds based on similarity to known protein structures (templates) (Marti-Renom et al. 2000) (see Materials and Methods). The corresponding sequence-structure alignments were refined and used to generate three-dimensional models of the nup domains, followed by evaluation of the models. Our analyses predicted that every nup in the yNup84/vNup107–160 subcomplex consists of a β-propeller domain, an α-solenoid domain, or both (Figure 1; Table 1). β-propellers contain several blades arranged radially around a central axis, each blade consisting of a four-stranded antiparallel β-sheet; α-solenoid domains are composed of numerous pairs of antiparallel α-helices stacked to form a solenoid (Figure 1) (Neer et al. 1994; Andrade et al. 2001a; Andrade et al. 2001b). While we have not defined the precise details of each domain, such as the exact shapes and numbers of propeller blades and solenoid repeats, the overall fold assignments for each nup are clear. These predictions indicate that yNup84, yNup85, and yNup145C all mainly consist of an α-solenoid domain, whereas yNup120 and yNup133 contain both an amino-terminal β-propeller and a large carboxyl-terminal α-solenoid region. Both ySec13 and ySeh1 are predicted to be almost entirely single-domain β-propellers of six and seven blades, respectively. These latter two proteins fall into the well-conserved class of tryptophan/aspartic acid (WD) repeat-containing β-propeller proteins. For both proteins, homology with the WD-repeat β-propellers has been reported previously (Saxena et al. 1996; Siniossoglou et al. 1996; Yu et al. 2000) and is confirmed here.

**Table 1:**
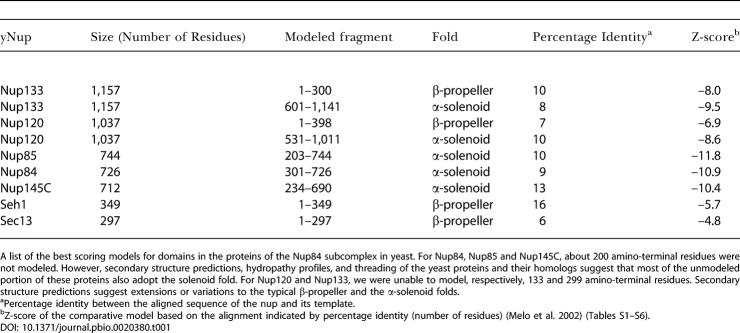
Nup84 Subcomplex Proteins are Composed of Two Fold Types

A list of the best scoring models for domains in the proteins of the Nup84 subcomplex in yeast. For Nup84, Nup85 and Nup145C, about 200 amino-terminal residues were not modeled. However, secondary structure predictions, hydropathy profiles, and threading of the yeast proteins and their homologs suggest that most of the unmodeled portion of these proteins also adopt the solenoid fold. For Nup120 and Nup133, we were unable to model, respectively, 133 and 299 amino-terminal residues. Secondary structure predictions suggest extensions or variations to the typical β-propeller and the α-solenoid folds

^a^Percentage identity between the aligned sequence of the nup and its template

^b^Z-score of the comparative model based on the alignment indicated by percentage identity (number of residues) (Melo et al. 2002) (Tables S1–S6)

**Figure 2:**
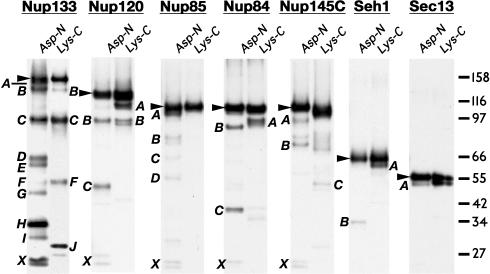
Proteolytic Domain Map of the Yeast Nup84 Subcomplex Proteins Immunoblots of limited proteolysis digests for Protein A-tagged versions of each of the seven nups in the yNup84 subcomplex. Each protein is detected via its carboxyl-terminal tag; thus, all the fragments visualized are amino-terminal truncations (except for the full length proteins, which are indicated by arrowheads). The fragments of the Asp-N and Lys-C protease digests depicted in Figure 2 are labeled with letters (A, B, C…) that correspond to those in Table 2, and the terminal Protein A fragments are labeled with an X (the Protein A tag is resistant to proteolysis). The sizes of marker proteins are indicated in kilodaltons (kDa) to the right of the gel.

**Figure 3:**
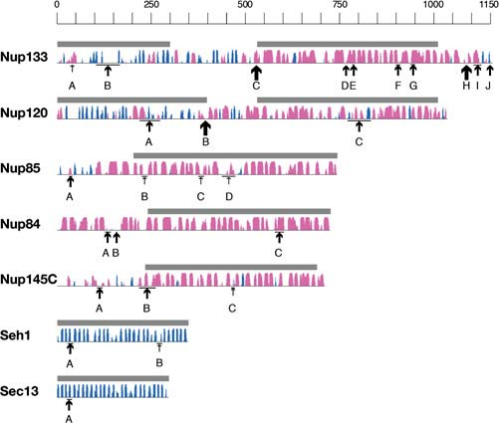
Predicted Secondary Structure Maps of the Nup84 Subcomplex Proteins Thin horizontal lines represent the primary sequence of each protein; secondary structure predictions are shown as columns above each line for β-strands (β-propellers; cyan) and α-helices (α-solenoids; magenta). The height of the columns is proportional to the confidence of the secondary structure prediction (McGuffin et al. 2000). The modeled regions are indicated above each sequence by horizontal dark bars, corresponding to the models in Figure 1. Proteolytic cleavage sites are identified by small, medium, and large arrows for weak, medium, and strong susceptibility sites, respectively. Where necessary, uncertainties in the precise cleavage positions are indicated above the arrows by horizontal bars.

We support our fold assignments using four considerations (Figure 2; Tables 1 and S1–S7). First, both fold assignment programs returned their predictions with highly significant scores (Tables S1–S7), and they predominantly assigned only the two predicted folds out of the approximately 1,000 different known fold types (Tables S1–S7) (Orengo et al. 1997). Moreover, while there are numerous variations corresponding to different proteins within each predicted fold type, the two different methods used for fold recognition often selected the same template proteins (Tables S1–S7). Second, the evaluation of the atomic model for each nup was statistically significant when compared against the best models generated for random sequences of identical amino acid composition and length; all the nup models were at least six standard deviations away from the mean score of the random models (Figure S1; Tables 1 and S1–S7) (Melo et al. 2002). Third, secondary structure predictions from amino acid sequences alone indicate that all seven nups consist mainly of repetitive structures that largely match the secondary structures observed in their corresponding three-dimensional models (Figure 3 and Figure S2). The agreement ranges from 58% to 87% of the residues for a three-state assignment (helix, strand, other). This agreement is the maximum possible level of consistency, given the approximately 75% accuracy of the secondary structure prediction methods (Koh et al. 2003).

**Table 2:**
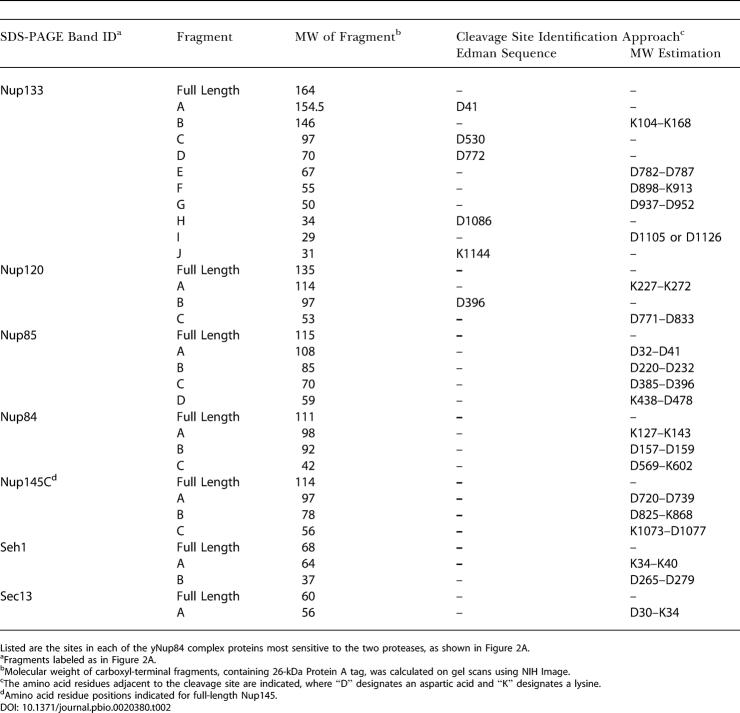
Proteolytically Sensitive Sites of yNup84 Subcomplex Proteins

Listed are the sites in each of the yNup84 complex proteins most sensitive to the two proteases, as shown in Figure 2A

^a^Fragments labeled as in Figure 2A

^b^Molecular weight of carboxyl-terminal fragments, containing 26-kDa Protein A tag, was calculated on gel scans using NIH Image

^c^The amino acid residues adjacent to the cleavage site are indicated, where “D” designates an aspartic acid and “K” designates a lysine

^d^Amino acid residue positions indicated for full-length Nup145

Finally, we provide direct biochemical evidence in support of our fold assignments, using proteolytic mapping of domain boundaries and loop locations in the seven nups (see Figure 2). Tagged nups were purified from yeast extracts and incubated with the endoproteinases Asp-N (which hydrolyzes peptide bonds at the amino side of aspartic acid) or Lys-C (which hydrolyzes peptide bonds at the carboxylic side of lysines) while still attached to the magnetic beads via their proteolytically resistant tags. After digestion, proteolytic fragments that remained attached to the beads were separated by SDS-PAGE, and cleavage sites were determined either by molecular weight estimation of the fragments or by amino-terminal Edman sequencing (Table 2). The regions predicted to form β-propellers were, as expected, extremely resistant to proteolysis (see Figure 2) (Kirchhausen and Harrison 1984; Saxena et al. 1996). On the whole, the predicted α-solenoid regions were also resistant to proteolysis, although less so than the β-propellers. However, the major cleavages were found toward the end of the predicted α-solenoid domains, even in the most susceptible nup (yNup133). Strikingly, the strongest cleavages generally occurred in the border regions between the predicted domains, as is particularly evident for yNup133 and yNup120 (Figure 3). Hence, in every case, the regions that we predicted to form compact folded structures were proteolytically resistant, and the predicted linkers between these domains were proteolytically sensitive. This correlation provides support for all seven of our structural models. In addition, circular dichroism and Fourier transform infrared spectra reported for Nup85 are in agreement with our predictions, indicating a composition characteristic of α-solenoids (approximately 50% α-helical, 23% loops, 5% turns, and 10% β-sheet) (Hirano et al. 1990; Denning et al. 2003). We expect our findings will spur efforts to determine the detailed atomic structures of nups; the rapid proteolytic domain mapping and molecular modeling techniques we have utilized here should aid these efforts.

Having established the domain folds for the yNup84 subcomplex, we also assigned domain folds in its vertebrate (i.e., human) and plant (i.e., *Arabidopsis*) homologs. All seven nups from both human and *Arabidopsis* yielded identical domain fold assignments to their yeast counterparts (Table S7), despite low primary sequence conservation among them (Suntharalingam and Wente 2003). These findings indicate that the overall architecture of the yNup84/vNup107–160 subcomplex has been preserved throughout the eukaryotes. Hence, the yNup84/vNup107–160 subcomplex, which contributes nearly one-quarter of the mass of the NPC, is composed in the main of repetitive β-propellers and α-solenoids; taken together with other repetitive domain nups (such as the FG repeat nups), this suggests that a significant percentage of the NPC's bulk is composed of protein repeats (Rout and Aitchison 2001; Suntharalingam and Wente 2003).

To gain insight into the function and origin of the yNup84/vNup107–160 subcomplex, we asked whether there are other known subcomplexes that share similar compositions and fold arrangements. A search of the entire SwissProt/TrEMBL database for entries that contain an amino-terminal β-propeller followed by an α-solenoid revealed that this specific architectural combination is absent from both bacteria and archaebacteria, and is found only in eukaryotic proteins, whose role (where known) is as components either of coated vesicles or of the yNup84/vNup107–160 subcomplex. Thus, the clathrin heavy chain, a major component of clathrin-coated vesicles, appears remarkably similar in domain architecture (ter Haar et al. 1998; Kirchhausen 2000b) to both yNup120/vNup160 and yNup133/vNup133. All three proteins are composed of an amino-terminal β-propeller followed by an extended α-solenoid. Proteolysis of assembled clathrin cages leads to the release of an amino-terminal fragment of 52–59 kDa (Kirchhausen and Harrison 1984). This result is similar to our domain mapping results, where the proteolysis of yNup120 and yNup133 resulted in amino-terminal fragments of 45 kDa and 60 kDa, respectively. Strikingly, one component of the yNup84/vNup107–160 subcomplex, ySec13/vSec13R, is also a known vesicle-coating protein. Similarly, ySeh1/vSec13L, a close homolog of ySec13/vSec13R, is also associated with both the yNup84/vNup107–160 subcomplex and the vesicle-coating proteins (Siniossoglou et al. 1996; Kirchhausen 2000b; Cronshaw et al. 2002; Gavin et al. 2002; Harel et al. 2003). Together, these results point to an intimate connection between vesicle-coating complexes and the yNup84/vNup107–160 subcomplex.

**Figure 4:**
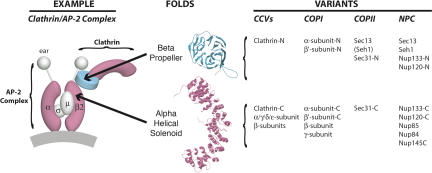
The Nup84 Complex and Coated Vesicles Share a Common Architecture A diagram showing the organization of the clathrin/AP-2 coated vesicle complex is shown at left; the positions of clathrin and the adaptin AP-2 large subunits (α, β2 plus “ear” domains) and small subunits (σ, μ) are indicated. β-propeller regions are colored cyan, α-solenoid regions are colored magenta, and sample ribbon models for each fold are shown in the center. The variants of each fold that are found as domains in major components of the three kinds of vesicle-coating complexes and the yNup84 subcomplex are listed on the right. The -N and -C indicate amino-terminal and carboxyl-terminal domains, respectively. The classification of these domains is based on X-ray crystallography data (clathrin, α-adaptin, β2-adaptin [PDB codes 1gw5, 1bpo, 1b89 (ter Haar et al. 1998; Collins et al. 2002)]), by the detailed homology modeling presented here (yNup84 complex proteins; ySec13 also in Saxena et al. [1996]), or by sequence homology or unpublished secondary structure prediction and preliminary analyses (COPI I (sec31) complex proteins [Schledzewski et al. 1999], Sec31).

In clathrin-coated vesicles, clathrin is attached via its amino-terminal domain to an adaptin complex. There are four types of adaptin complexes, all made of two large subunits that wrap around two small subunits. The bulk of each large subunit is made of an α-solenoid trunk (Figure 4) (Collins et al. 2002; Evans and Owen 2002). Similarly, the bulk of yNup84/vNup107, yNup85/vNup75, and yNup145C/vNup96 are also composed of α-solenoid trunks. Hence, the yNup84/vNup107–160 subcomplex resembles the clathrin/adaptin complex, in that the clathrin-like yNup120/vNup160 and yNup133/vNup133 are attached to the adaptin-like proteins yNup84/vNup107, yNup85/vNup75, and yNup145C/vNup96. This resemblance is further strengthened by our observation that the preferred templates for modeling the α-solenoid domains in the yNup84/vNup107–160 subcomplex were derived from proteins in vesicle coating complexes (Figure S1; Tables S1–S7).

Our analyses showed that the yNup84/vNup107–160 subcomplex and all three major classes of vesicle coating complexes can be linked together through their common architecture. As summarized in Figure 4, these similarities include both previously reported relationships (e.g., between the clathrin/adaptin complexes and the COPI complexes) (Schledzewski et al. 1999), and previously unsuspected relationships (e.g., between the COPII component Sec31 [Salama et al. 1997; Shugrue et al. 1999; Belden and Barlowe 2001; Boehm and Bonifacino 2001; Lederkremer et al. 2001] and clathrin).

The common architecture of the yNup84/vNup107–160 subcomplex and all three major classes of vesicle-coating complexes suggests that all of these complexes have common function in curving membranes. There is, in fact, circumstantial evidence for a role of the yNup84/vNup107–160 subcomplex in the establishment and maintenance of pore membrane curvature. Members of this complex, when disrupted in yeast, cause the uniformly distributed NPCs to cluster into patches in the plane of the NE (Siniossoglou et al. 1996; Siniossoglou et al. 2000; Ryan and Wente 2002; Teixeira et al. 2002), suggesting that impairment of yNup84 subcomplex function results in a suboptimal interaction of the NPC with its surrounding nuclear membranes.

## Discussion

As shown here, protein structure modeling is particularly useful in uncovering potential evolutionary and functional relationships that are refractory to classical approaches based on comparison of protein sequences alone. Our results show that clathrin/adaptin complexes, COPI complexes, COPII complexes, and the yNup84/vNup107–160 subcomplex all share a common molecular architecture. This commonality could have arisen by either convergent or divergent evolutionary pathways.

In a convergent pathway, β-propeller and α-solenoid folds could have been independently utilized by both NPCs and vesicle-coating complexes at different stages of eukaryotic evolution. This possibility is supported by the high abundance of both fold types in eukaryotic genomes (which could potentially make their fusion in proteins or complexes relatively frequent) (Yanai et al. 2002) and the low sequence similarities between proteins of the NPC and vesicle coating complexes (which may suggest that they are not related).

In a divergent pathway, NPCs and vesicle-coating complexes share these folds because both complex types could have originated from a common ancestor. In this scenario, a single “protocoatomer” would have been the progenitor for numerous vesicle coating complexes, as well as the yNup84/vNup107–160 subcomplex. Several lines of evidence support this latter hypothesis. First, the most confident models of the yNup84/vNup107–160 subcomplex proteins are based on structures of coated vesicle proteins (Figure S1; Tables S1–S7). Second, the particular arrangement of an amino-terminal β-propeller followed by an α-solenoid appears to be unique to components of either vesicle coating complexes or of the yNup84/vNup107–160 subcomplex (Protocol S1). Third, the overall composition of both complex types is similar, being mainly composed of proteins containing comparable distributions of β-propellers and α-solenoids (Figure 4). Fourth, both vesicle coating complexes and NPCs apparently share a common function: the bending and stabilizing of curved membranes. Fifth, the yNup84/vNup107–160 subcomplex actually contains bona fide vesicle coat components, Sec13 and Seh1. In light of these considerations, we favor the “protocoatomer” hypothesis, in which the NPCs and vesicle-coating complexes arose by a process of divergent evolution.

**Figure 5:**
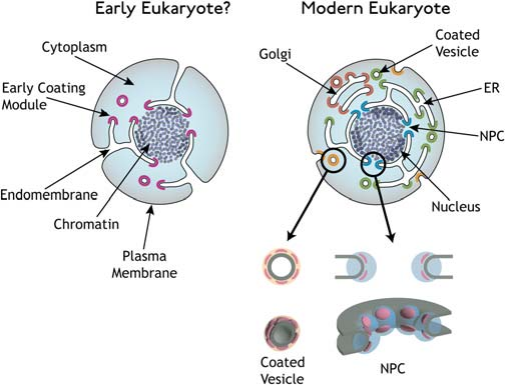
Proposed Model for the Evolution of Coated Vesicles and Nuclear Pore Complexes Early eukaryotes (left) acquired a membrane-curving protein module (purple) that allowed them to mold their plasma membrane into internal compartments and structures. Modern eukaryotes have diversified this membrane-curving module into many specialized functions (right), such as endocytosis (orange), ER and Golgi transport (green and brown), and NPC formation (blue). This module (pink) has been retained in both NPCs (right bottom) and coated vesicles (left bottom), as it is needed to stabilize curved membranes in both cases.

The lack of detectable sequence similarity between the proteins in the yNup84/vNup107–160 subcomplex and the coated vesicles is not surprising. Sequence comparisons of α-solenoid- and β-propeller-containing proteins suggest that these folds arose just before or around the time of the origin of eukaryotes, then rapidly duplicated and diversified (Cingolani et al. 1999; Smith et al. 1999; Andrade et al. 2001b). Both folds consist of repetitive structures, so the functional constraints on an individual repeat are weak, compared with the whole fold domain. It has been proposed that the robustness of these folds with respect to changes in their sequences permits their component repeats to individually lose their sequence similarity, eventually allowing the proteins they comprise to drift into new functions (Malik et al. 1997; Smith et al. 1999; Andrade et al. 2001a; Andrade et al. 2001b). Moreover, the lack of detectable sequence similarity for members of the same fold family is not necessarily an indicator of convergent evolution; obvious sequence similarities are often lost during long periods of evolution (e.g., FtsZ and tubulin or MreB and actin [Amos et al. 2004]). The divergent pathway is also consistent with the conservation among members of the syntaxin family (key components of the vesicular transport machinery), which points to a similar early origin and rapid diversification of the eukaryotic endomembrane system (Dacks and Doolittle 2002; Dacks and Field 2004). Based on these observations, we propose a single evolutionary origin for the structures maintaining both the endomembrane systems and the nucleus (Figure 5) over models suggesting separate or even endosymbiotic origins for these structures.

The current protocoatomer hypothesis posits that a simple coating module containing minimal copies of the two conserved folds evolved in protoeukaryotes as a mechanism to bend membranes into sharply curved sheets and invaginated tubules (Figure 5). The ability to so manipulate cell membranes represented a major evolutionary innovation that allowed, among other possibilities, the elaboration of internal membranes, phagotrophy, and endosymbiosis (Maynard Smith and Szathmâary 1997); the importance of this ability is underscored by the presence of numerous types of membrane-curving devices in modern eukaryotes. As with clathrin, the flexibility of the α-solenoid in this simple module enabled the formation of curved membranes of various sizes. In addition, the α-solenoid repeat structure, together with the repeats in the β-propeller fold, provided the coating module with a large binding area. These features allowed the membrane-curving module to polymerize and form a coat, as well as to interact with other membrane-associated proteins. The endomembranes and their membrane-coating modules subsequently evolved to become more elaborate and specialized, with the partitioning of different functions into separate, interconnected compartments such as the ER, the Golgi, and the nucleus (Figure 5), each with their own specialized set of coating modules.

In conclusion, we suggest that the progenitor of the NPC arose from a membrane-coating module that wrapped extensions of an early ER around the cell's chromatin. In this primitive NE, the coating modules would have originally formed the sharply curved membrane, creating large and freely permeable pores (Figure 5). These pores then closed to form the selectively permeable NPCs of modern eukaryotes (Rout et al. 2003). In doing so, they retained at their core a coating module as a relic of their evolutionary origins. This module, the yNup84/vNup107–160 subcomplex, may still serve to curve and stabilize the nuclear pore membrane in modern eukaryotes; as such, it would function as a key scaffold to form the NPCs, the portals of the nucleus. Our findings could thus provide an explanation for the origin of the nuclear pore complex (which until now has been a mystery) and may fill a significant gap in our understanding of the evolution of eukaryotes.

## Materials and Methods

### 

Only two domains in the seven nups have their folds assigned by sequence comparison to proteins of known structure (Saxena et al. 1996; Siniossoglou et al. 1996). Therefore, to assign folds for as many target domains comprising the yNup84/vNup107–160 subcomplex as possible, we applied a structure-based approach consisting of iterative detection of potential template structures, their alignment to the target sequence, model building, and model assessment (Marti-Renom et al. 2000). Secondary structure was predicted from sequence by the PredictProtein (Rost 1996) and PSI-Pred (McGuffin et al. 2000) servers.

#### Detection of potential template structures

For each of the seven yeast nups and representative homologs, potentially related known structures were detected by the mGenThreader (McGuffin and Jones 2003) and FUGUE (Shi et al. 2001) web servers (Tables S1–S7). Several other servers gave similar results (unpublished data). To find out whether or not mGenThreader frequently identifies the β-propeller and α-solenoid folds as false positives, we randomly selected 20 sequences of known structure from each one of the structural classes and submitted them to mGenThreader. Using the same parameters as in our analysis of the nups, only two of these 140 sequences were incorrectly predicted to contain β-propeller or α-solenoid folds (unpublished data). Thus, we estimate the false positives rate for the nup fold assignments based on mGenThreader alone to be approximately 1%–2%.

#### Alignment of the matched target-template pairs

The matches obtained in the previous step provided an operational definition of a domain. They were either accepted or refined by manual and automated alignment. Manual realignment relied on sequence conservation and secondary structure predictions by PROF (Rost 1996) and PSI-PRED (McGuffin et al. 2000). The automatic realignments were obtained by SALIGN (Marti-Renom et al. 2004) and T-Coffee (Notredame et al. 2000). In the last iteration, the alignments and the models were refined by MOULDER, a genetic algorithm method for iterative alignment, model building, and model assessment (John and Sali 2003).

#### Model building

For each alignment, an all-atom model was built by comparative modeling based on satisfaction of spatial restraints as implemented in MODELLER (Sali and Blundell 1993).

#### Model assessment.

The fold assignment, alignment, and model building were repeated by varying the domain boundaries, target sequences for modeling, template structures, and their alignments. The aim was to improve model assessment by statistical potentials of ProsaII (Sippl 1993) and DFIRE (Zhou and Zhou 2002), and by a composite model evaluation criterion (Melo et al. 2002; John and Sali 2003). The only importance of explicit model building in this analysis was to provide another semi-independent way to validate the fold assignments: If a model was assessed to have the correct fold, the initial fold assignment must have been correct. Beyond that, the models were not used.

#### Domain combination search.

To search for proteins that resemble the domain architecture of clathrin, we queried MODBASE (Pieper et al. 2004), our relational database of annotated comparative protein structure models, and Superfamily (Gough et al. 2001), a database of HMM-based structural assignments. Both databases assign folds to all available protein sequences that match at least one known protein structure. We first searched for any protein sequences that were matched to both β-propeller and α-solenoid structures. We used the broadest definitions of the β-propeller folds (b.66, b.67, b.68, b.69, b.70, for 4-, 5-, 6-, 7- and 8-bladed β-propellers, respectively) and α-solenoid folds (a.118) from the SCOP database (v1.65) (Lo Conte et al. 2002). In MODBASE, we found 95 proteins predicted to contain both β-propeller and α-solenoid domains (Protocol S1). Of these 95 proteins, 37 passed the following filters, ensuring clathrin-like characteristics: they had to be 800–2,000 residues long, the amino-terminal β-propeller domain had to be followed by a carboxyl-terminal α-solenoid domain, the β-propeller and α-solenoid domains each had to span at least 35% of the total length, and no other domain could be more than 25% of the total length. All of the 37 proteins were from eukaryotes. Their functions were assigned either as clathrin or unknown in the Swiss-Prot/TrEMBL database (O'Donovan et al. 2002). Similar results were obtained by querying the Superfamily database (Gough et al. 2001).

#### Proteolytic domain laddering.

Magnetic beads (2.8 μm Dynabeads M-270 Epoxy [#143.02; Dynal, Oslo, Norway]) were conjugated to rabbit IgG (#55944; ICN Biochemicals, Costa Mesa, California, United States) according to the manufacturer's instructions. Yeast cells carrying PrA-tagged versions of nups were grown and harvested as described previously (Rout et al. 2000). Cell pellets were frozen in liquid nitrogen and homogenized to a fine powder in a motorized grinder (#RM100; Retsch, Haan, Germany) continuously cooled with liquid nitrogen. The cell powder was thawed on ice and ten volumes of extraction buffer (20 mM HEPES [pH 7.4], 1.0% Triton X-100, 0.5% sodium deoxycholate, 0.3% sodium N-lauroyl-sarcosine, 0.1 mM MgCl_2_, 1 mM DTT, 1:500 protease inhibitor cocktail [#P-8340; Sigma, St. Louis, Missouri, United States]) were added to cells and homogenized at 4 °C with a Polytron (Kinematica, Littau-Luzerne, Switzerland). The cell lysate was clarified by centrifugation (2,000 *g* for 5 min at 4 °C). The magnetic beads were added to the extract to a ratio of about 8 × 10^9^ beads per g of cells. After incubation for 1 h at 4 °C, the beads were magnetically recovered. The beads were washed, resuspended in 50 μl of reaction buffer (according to the manufacturer's specifications), and Asp-N (#1420488; Roche, Basel, Switzerland) or Lys-C (#1420429; Roche) proteinase was added to give a weight ratio of 1:200 of proteinase to the tagged nup. After incubation at different time points at 37 °C, bead aliquots were removed and washed, and tagged fragments were eluted with 0.5 M NH_4_OH containing 0.5 mM EDTA. The eluant was vacuum-dried, resuspended in SDS-PAGE sample buffer, and separated on a 4%–12% bis-Tris gel (Invitrogen, Carlsbad, California, United States). Proteins were then either transferred electrophoretically to nitrocellulose or PVDF and probed with HRP-rabbit IgG (#011–0303-003; Jackson ImmunoResearch, West Grove, Pennsylvania, United States), or analyzed by amino-terminal Edman sequencing (Fernandez et al. 1994).

## Supporting Information

Figure S1 Model Score Versus Length The graphs plot the assessment score of the model (Melo Z-score) (Melo et al. 2002) versus the model size, for the "non-MOULDER" models in Tables S2–S6. The red circles indicate the entries in Table 1 in the main text of the paper. Because the Z-score depends on the number of residues in the model, the smallest model with the highest Z-score was considered most significant.
(87 KB DOC).

Figure S2 Agreement between Predicted and Modeled Secondary Structure The secondary structure predicted from sequence by PROF (Rost and Liu 2003) and PSI-Pred (McGuffin et al. 2003) is compared to the secondary structure observed in the three-dimensional models presented in Table S1 (“…” represents regions that are not modeled). The numbers above the predicted secondary structures correspond to the confidence score returned by the servers. Current secondary structure prediction methods based on multiple alignments correctly predict the secondary structure state for 70%–80% of residues (in a three-state prediction) (Eyrich et al. 2001). Since the random prediction would predict only about 30% of the residues correctly, the fact that our predictions match the assignments at 58%–87% level is highly suggestive, supporting our fold assignments. A representative example, Nup85, is shown here. For the visualization of all the Nups, see the additional information web page (http://salilab.org/damien/NPC/).
(47 KB DOC).

Protocol S1 List of Proteins Modeled as β-Propeller and α-Solenoid Domains in ModBase (42 KB DOC).

Table S1 Modeling Results for Yeast Nup84 Complex Proteins I (yNup133) (491 KB DOC).

Table S2 Modeling Results for Yeast Nup84 Complex Proteins II (yNup133) (101 KB DOC).

Table S3 Modeling Results for Yeast Nup84 Complex Proteins III (yNup133) (115 KB DOC).

Table S4 Modeling Results for Yeast Nup84 Complex Proteins IV (yNup133) (132 KB DOC).

Table S5 Modeling Results for Yeast Nup84 Complex Proteins V (yNup133) (124 KB DOC).

Table S6 Modeling Results for Yeast Nup84 Complex Proteins (yNup133) (93 KB DOC).

Table S7 Modeling Results for Human and Plant Nup84 Complex Proteins (yNup133) (144 KB DOC).

### Accession Numbers

Uniprot (Apweiler et al. 2004) accession numbers (http://www.pir.uniprot.org) for proteins discussed in this paper are as follows. Yeast: ySeh1 (P53011), ySec13 (Q04491), yNup84 (P52891), yNup85 (P46673), yNup120 (P35729), yNup133 (P36161), and yNup145C (P49687). Human: vSec13 l (Q96EE3), vSec13R (P55735), vNup107 (P57740), vNup75 (Q9BW27), vNup160 (Q12769), vNup133 (Q8WUM0), and vNup96 (P52948).

## Abbreviations

COP: coat protein
ER: endoplasmic reticulum
NE: nuclear envelope
NPC: nuclear pore complex
nup: nucleoporin
WD: tryptophan/aspartic acid
