# Correction: Components of Coated Vesicles and Nuclear Pore Complexes Share a Common Molecular Architecture

**DOI:** 10.1371/journal.pbio.0030080

**Published:** 2005-02-15

**Authors:** Damien Devos, Svetlana Dokudovskaya, Frank Alber, Rosemary Williams, Brian T Chait, Andrej Sali, Michael P Rout

In *PLoS Biology*, volume 2, issue 12.

10.1371/journal.pbio.0020380

In Materials and Methods, the sentence “The magnetic beads were added to the extract to a ratio of about 8 ×10^9^ beads per g of cells” contains an error. The correct amount of beads is 8 × 10^8^, i.e., ten times less.

Published February 15, 2005

