# Supplementary material for: Components of Coated Vesicles and Nuclear Pore Complexes Share a Common Molecular Architecture
Source: PLoS Biol. 2004 Nov 2;2(12):e380. doi: 10.1371/journal.pbio.0020380 (PMC524472; doi:10.1371/journal.pbio.0020380)
Supplement: Figure S1 — The graphs plot the assessment score of the model (Melo Z-score) (Melo et al. 2002) versus the model size, for the "non-MOULDER" models in Tables S2–S6. The red circles indicate the entries in Table 1 in the main text of the paper. Because the Z-score depends on the number of residues in the model, the smallest model with the highest Z-score was considered most significant. (87 KB DOC). [file pbio.0020380.sg001.doc]

### Supplementary Figure S1. Model score *versus* length

The graphs plot the assessment score of the model (Melo Z-score) (Melo et al. 2002) versus the model size, for the "non-MOULDER" models in Supplementary Tables 2-6. The red circles indicate the entries in Table 1 in the main text of the paper. Because the Z-score depends on the number of residues in the model, the smallest model with the highest Z-score was considered most significant.

a) Nup133

-9

-8

-7

-6

-5

-4

-3

-2

-1

0

250

300

350

400

450

500

550

600

650

Model size

Z-score

b) Nup120

-8

-7

-6

-5

-4

-3

-2

-1

0

350

400

450

500

550

600

650

Model size

Z-score

c) Nup85

-9

-8

-7

-6

-5

-4

-3

-2

-1

0

520

540

560

580

600

620

640

660

Model size

Z-score

d) Nup84

-9

-8

-7

-6

-5

-4

-3

-2

-1

0

350

400

450

500

550

600

650

700

750

Model size

Z-score

e) Nup145C

-9

-8

-7

-6

-5

-4

-3

350

400

450

500

550

600

650

700

750

Model size

Z-score
