# Supplementary material for: Components of Coated Vesicles and Nuclear Pore Complexes Share a Common Molecular Architecture
Source: PLoS Biol. 2004 Nov 2;2(12):e380. doi: 10.1371/journal.pbio.0020380 (PMC524472; doi:10.1371/journal.pbio.0020380)
Supplement: Figure S2 — The secondary structure predicted from sequence by PROF (Rost and Liu 2003) and PSI-Pred (McGuffin et al. 2003) is compared to the secondary structure observed in the three-dimensional models presented in Table S1 (“…” represents regions that are not modeled). The numbers above the predicted secondary structures correspond to the confidence score returned by the servers. Current secondary structure prediction methods based on multiple alignments correctly predict the secondary structure state for 70%–80% of residues (in a three-state prediction) (Eyrich et al. 2001). Since the random prediction would predict only about 30% of the residues correctly, the fact that our predictions match the assignments at 58%–87% level is highly suggestive, supporting our fold assignments. A representative example, Nup85, is shown here. For the visualization of all the Nups, see the additional information web page (http://salilab.org/damien/NPC/). (47 KB DOC). [file pbio.0020380.sg002.doc]

Supplementary Figure 2. Agreement between predicted and modeled secondary structure for the Nup85 protein

In the following pages, the secondary structure predicted from sequence by PROF (Rost and Liu 2003) and PSI-Pred (Jones 1999) is compared to the secondary structure observed in the three-dimensional models presented in Supplementary Table I (“…” represents regions that are not modeled). The numbers above the predicted secondary structures correspond to the confidence score returned by the servers.

Current secondary structure prediction methods based on multiple alignments correctly predict the secondary structure state for 70-80% of residues (in a 3 state prediction) (Eyrich et al. 2001). Since the random prediction would predict only approx. 30% of the residues correctly, the fact that our predictions match the assignments at 58-87% level is highly suggestive, supporting our fold assignments at a sufficiently high level to be worth mentioning in the manuscript. It certainly does not support the view that fold assignments are incorrect.

A representative example, Nup85 is shown here. For the visualization of all the Nups, see the additional information web page (http://salilab.org/~damien/NPC/).

........10........20........30........40........50

Nup85 MELSPTYQTERFTKFSDTLKEFKIEQNNEQNPIDPFNIIREFRSAAGQLA

97776531011124789999999999724687647999999999986467

PSI-Pred --------HHHHHHHHHHHHHHHHHHHH------HHHHHHHHHHHHHHHH

90244425132013467888877741466787225788887775565443

PROF --------HHHHHHHHHHHHHHHHHH--------HHHHHHHHHHHHHHHH

Model ..................................................

........60........70........80........90.......100

LDLANSGDESNVISSKDWELEARFWHLVELLLVFRNADLDLDEMELHPYN

64068730123366666688899999999860203541103340134540

HH-----HHHHHHHHHHHHHHHHHHHHHHHHH--------HHHH-----H

22236642222445677778887787888765201132010102102424

HHH-----HHHHHHHHHHHHHHHHHHHHHHHHHH----------------

..................................................

.......110.......120.......130.......140.......150

SRGLFEKKLMQDNKQLYQIWIVMVWLKENTYVMERPKNVPTSKWLNSITS

48888999841377899999999997305402100688620000010011

HHHHHHHHHH---HHHHHHHHHHHHHHH---HHH------EE-EEEE---

15677887521660377776778778742322110113553200000001

-HHHHHHHHH---HHHHHHHHHHHHHHHHHHHHHH---------------

..................................................

.......160.......170.......180.......190.......200

GGLKSCDLDFPLRENTNVLDVKDKEEDHIFFKYIYELILAGAIDEALEEA

44210488887641000340454478999999999998502579999999

-------------------HHHHHHHHHHHHHHHHHHHH---HHHHHHHH

22322466755000024455335567888898888875236636788887

-----------------------HHHHHHHHHHHHHHHH---HHHHHHHH

..................................................

.......210.......220.......230.......240.......250

KLSDNISICMILCGIQEYLNPVIDTQIANEFNTQQGIKKHSLWRRTVYSL

74663678987435567725887443320002445751578999999998

HH--HHHHHHHH--HHHHH------------------HHHHHHHHHHHHH

54042568876402212026442221222115434665246778877764

HH---HHHHHHHH--HHH--------------------HHHHHHHHHHHH

...................................---HHHHHHHHHHHH

.......260.......270.......280.......290.......300

SQQAGLDPYERAIYSYLSGAIPNQEVLQYSDWESDLHIHLNQILQTEIEN

76418963788989998505233210223788889999999999999999

HHH----HHHHHHHHHHHH------------HHHHHHHHHHHHHHHHHHH

21037782235455544134322100123564678888887543345566

HH-----HHHHHHHHHHH-------------HHHHHHHHHHHHHHHHHHH

-HHHHHHHHHHHHHHH---------------HHHHHEHHHHHHHHHHHHH

.......310.......320.......330.......340.......350

YLLENNQVGTDELILPLPSHALTVQEVLNRVASRHPSESEHPIRVLMASV

99743786301102230466888899999998630101253257888887

HHHH------HHHHHHH------HHHHHHHHHHH-------HHHHHHHHH

76620454402311556543556688888775301012011246777876

HHHH------HHH----------HHHHHHHHHHH-HHHH--HHHHHHHHH

HHHH-----HHHHHHHHH--HHHHHHHHHHHHHH---HHHHHHHHHHHH-

.......360.......370.......380.......390.......400

ILDSLPSVIHSSVEMLLDVVKGTEASNDIIDKPYLLRIVTHLAICLDIIN

40220256667889997631443456787886413446666557865326

HH---HHHHHHHHHHHHHH--------------HHHHHHHHHHHHHHH--

76434666642377677752132113444461467777888888887407

HHHHHHHHHHHHHHHHHHHH------------HHHHHHHHHHHHHHHHH-

------HHHHHHHHHHHHH------------------HHHHHHHHHHHH-

.......410.......420.......430.......440.......450

PGSVEEVDKSKLITTYISLLKLQGLYENIPIYATFLNESDCLEACSFILS

63400100589999999999866970478899964484233899999998

-----HHHHHHHHHHHHHHHHH----HHHHHHHHH--HHHHHHHHHHHHH

32100101357888888888611520026778752252036888888886

---HHHH-HHHHHHHHHHHHHH-----HHHHHHHH---HHHHHHHHHHHH

--HHHHHHHHHHHHHHHHHH---------HHHHHHHHH--HHHHHHHHHH

.......460.......470.......480.......490.......500

SLEDPQVRKKQIETINFLRLPASNILRRTTQRVFDETEQEYSPSNEISIS

51671478999999996187467777877777630462342002221024

HH---HHHHHHHHHHHHH---HHHHHHHHHHHHH---HHHHHHHHHH---

40260012577888874078735676677886543211015766432201

H-----HHHHHHHHHHH----HHHHHHHHHHHHHHHHH------------

HHHHH-----HHHHHHHH--------HHHHHHH----------------H

.......510.......520.......530.......540.......550

FDVNNIDMHLIYGVEWLIEGKLYVDAVHSIIALSRRFLLNGRVKALEQFM

67776777656533200266889999999999999997157058999997

----HHHHHHHHHHH-----HHHHHHHHHHHHHHHHHHH--HHHHHHHHH

34331104443212100134221357888888888876510368888763

-----HHHHHHHHHHHEE-----HHHHHHHHHHHHHHHHH-HHHHHHHHH

HHHHHHHHHHHH------HHHHHHHHHHHHHHHHHHHHH-----HHHHHH

.......560.......570.......580.......590.......600

ERNNIGEICKNYELEKIADNISKDENEDQFLEEITQYEHLIKGIREYEEW

21776688763200101367765477216898741279999999889999

HH--HHHHHHHHHH-------------HHHHHHHHHHHHHHHHHHHHHHH

10572466544323102134773200134577777788888867655666

H---HHHHHHHHHHHH----------HHHHHHHHHHHHHHHHHHHHHHHH

HHHHHHHHHHHHHHHH--HHHHHHHHHHHHHHHH----HHHHHHHHHHHH

.......610.......620.......630.......640.......650

QKSVSLLSSESNIPTLIEKLQGFSKDTFELIKTFLVDLTSSNFADSADYE

99833688765507899999888799999877764323320468988899

HHHH--------HHHHHHHHHHHHHHHHHHHHHHHHHHHH------HHHH

67430035665661255530200055788877777622442035703467

HHHH----------HHHHHH---HHHHHHHHHHHHHHHHHHH---HHHHH

HHHHHH--------HHHHHHHHHHH--HHHHHHHHHHHHHHHH---HHHH

.......660.......670.......680.......690.......700

ILYEIRALYTPFLLMELHKKLVEAAKLLKIPKFISEALAFTSLVANENDK

99999986479999999999863577615542068899999987430001

HHHHHHHHHHHHHHHHHHHHHHHHHHHH-----HHHHHHHHHHHHHH--H

88888740106778777777765553303520256767788664323002

HHHHHHHH--HHHHHHHHHHHHHHHHH----HHHHHHHHHHHHHHHHHHH

HHHHHHH-----HHHHHHHHHHHH-------HHHHHHHHHHHHH------

.......710.......720......

IYLLFQSSGKLKEYLDLVARTATLSN

23430351789999999998863169

HHHHH---HHHHHHHHHHHHHHH---

43320024544888888876400368

HHHHH-----HHHHHHHHHHHH----

------------HHHHHHHHHHH---
