# Supplementary material for: Components of Coated Vesicles and Nuclear Pore Complexes Share a Common Molecular Architecture
Source: PLoS Biol. 2004 Nov 2;2(12):e380. doi: 10.1371/journal.pbio.0020380 (PMC524472; doi:10.1371/journal.pbio.0020380)
Supplement: Protocol S1 — (42 KB DOC). [file pbio.0020380.sd001.doc]

### Supplementary List of proteins modeled as β-propeller and α-solenoid domains in ModBase

In the following, we list the proteins detected in ModBase (Pieper et al. 2004) modeled as a β-propeller and a α-solenoid domain. The listed codes are either Swiss-Prot or GeneBank (GI).

21294710 (GI)

31247948 (GI)

32446366 (GI)

32447475 (GI)

33438248 (GI)

O42900

O96221

P11442

P22137

P22219

P25870

P29742

P34574

P38873

P49951

P53675

P87141

Q00610

Q10161

Q17901

Q21106

Q22830

Q23669

Q39834

Q7PQY9

Q7Q106

Q7Q7G1

Q7QD70

Q7QGJ2

Q7R4L8

Q7RNG3

Q7SHV2

Q7SI31

Q80T73

Q80U89

Q86JF2

Q86TF2

Q8BND3

Q8C794

Q8C8U5

Q8CD77

Q8CVE4

Q8I1M1

Q8I1Q1

Q8I3S3

Q8I5L6

Q8IHL0

Q8JZZ2

Q8K3R5

Q8K4Q0

Q8MQT9

Q8N102

Q8N122

Q8NE11

Q8NEZ3

Q8NFQ0

Q8NJL6

Q8TC06

Q8UUQ9

Q8UUR1

Q8WT27

Q8X1X8

Q8YMU3

Q93YQ1

Q95Z06

Q961J4

Q96RY7

Q99570

Q9C6U0

Q9C982

Q9CA00

Q9ESD4

Q9ESE1

Q9H2U4

Q9LR78

Q9LZW9

Q9M0E5

Q9NV87

Q9P2L0

Q9P2P3

Q9P5N6

Q9SJF0

Q9SR85

Q9SRM1

Q9UBV2

Q9UPW4

Q9UVG6

Q9V8C7

Q9VHH2

Q9VPR0

Q9W040

Q9W437

Q9XTJ1

Q9Y5L9

Q9Z2G6
