# Supplementary material for: Components of Coated Vesicles and Nuclear Pore Complexes Share a Common Molecular Architecture
Source: PLoS Biol. 2004 Nov 2;2(12):e380. doi: 10.1371/journal.pbio.0020380 (PMC524472; doi:10.1371/journal.pbio.0020380)
Supplement: Table S1 — (491 KB DOC). [file pbio.0020380.st001.doc]

### Supplementary Table 1. Modeling results summary

The following annotations are used: mGTh, mGenThreader (McGuffin and Jones 2003); Fugue (Shi et al. 2001); Moulder# indicates the rank order of the MOULDER model (John and Sali 2003); SALIGN module of MODELLER (Marti-Renom et al. 2004); Prosa II Z-score (Sippl 1993), Dfire (Zhou and Zhou 2002); GA341 score (from 0 for models that tend to have an incorrect fold to 1 for models that tend to have at least the correct fold) and Melo Z-score (Melo et al. 2002).

| ***Nups*** | ***Size*** | ***Modeled***  ***fragment*** | ***Method*** | ***Id*** | ***Template***  ***Size*** | ***fragment*** | ***%id*** | ***GA341***  ***Score*** | ***Melo***  ***Z-score*** |
| --- | --- | --- | --- | --- | --- | --- | --- | --- | --- |
| Nup133 | 1157 | 1-300 | Moulder3 | 1erjA | 350 | 328-710 | 10 | 1.00 | -8.04 |
| Nup133 | 1157 | 601-1141 | Moulder0 | 1gw5A | 584 | 9-508 | 8 | 1.00 | -9.53 |
|  |  |  |  |  |  |  |  |  |  |
| Nup85 | 744 | 203-744 | Moulder2 | 1gw5A | 584 | 9-508 | 10 | 1.00 | -11.84 |
|  |  |  |  |  |  |  |  |  |  |
| Nup84 | 726 | 322-715 | Moulder4 | 1ee4A | 423 | 87-509 | 10 | 1.00 | -10.92 |
|  |  |  |  |  |  |  |  |  |  |
| Nup120 | 1037 | 1-398 | Moulder1 | 1pguB | 608 | 18-388 | 7 | 1.00 | -6.94 |
| Nup120 | 1037 | 531-1011 | Moulder1 | 1ee4A | 423 | 87-509 | 10 | 1.00 | -8.63 |
|  |  |  |  |  |  |  |  |  |  |
| Nup145C | 712 | 234-690 | Moulder2 | 1bk5A | 422 | 89-509 | 13 | 1.00 | -10.38 |
