# Supplementary material for: Components of Coated Vesicles and Nuclear Pore Complexes Share a Common Molecular Architecture
Source: PLoS Biol. 2004 Nov 2;2(12):e380. doi: 10.1371/journal.pbio.0020380 (PMC524472; doi:10.1371/journal.pbio.0020380)
Supplement: Table S2 — (101 KB DOC). [file pbio.0020380.st002.doc]

### Supplementary Table 2. Nup133 modeling results

The following annotations are used: mGTh, mGenThreader (McGuffin and Jones 2003); Fugue (Shi et al. 2001); Moulder# indicates the rank order of the MOULDER model (John and Sali 2003); SALIGN module of MODELLER (Marti-Renom et al. 2004); Prosa II Z-score (Sippl 1993), Dfire (Zhou and Zhou 2002); GA341 score (from 0 for models that tend to have an incorrect fold to 1 for models that tend to have at least the correct fold) and Melo Z-score (Melo et al. 2002).

| ***Nups*** | ***Prt size*** | ***Modeled***  ***fragment*** | ***Origin*** | ***Template*** | | | ***%id*** | ***Th Score*** | ***ProsaII Z-score*** | | ***GA341***  ***Score*** | ***Melo***  ***Z-score*** | ***Dfire*** |
| --- | --- | --- | --- | --- | --- | --- | --- | --- | --- | --- | --- | --- | --- |
|  |  |  |  | ***Id*** | ***Size*** | ***fragment*** |  |  | ***Model*** | ***Template*** |  |  |  |
| Nup133 | 1157 | 77-532 | mGTh | 1eut | 601 | 1-478 | 10.7 | 0.062 | -3.58 | -10.13 | 0.33 | -5.07 |  |
| Nup133 | 1157 | 1-300 | mGTh | 1erjA | 350 | 328-710 | 10 | 0.071 | -4.75 | -10.01 | 1.00 | -7.93 | -315.58 |
| Nup133 | 1157 | 133-472 | mGTh | 1fnf | 368 | 1142-1509 | 9 | 0.171 | -4.08 | -11.72 | 0.01 | -2.68 |  |
| Nup133 | 1157 | 1-532 | mGTh | 1k32 | 1023 | 399-943 | 6 | 0.125 |  |  | 1.00 | -5.21 |  |
| Nup133 | 1157 | 1-496 | mGTh | 1gof | 639 | 121-639 | 6 | 0.117 |  |  | 0.85 | -2.28 |  |
| Nup133 | 1157 | 1-420 | Fugue | 1jju | 489 | 1-480 | 14 | 3.04 |  |  | 0.36 | -4.87 |  |
| Nup133 | 1157 | 1-420 | Fugue | 1gotB | 339 | 2-340 | 8 | 2.37 |  |  | 1.00 | -4.36 |  |
| Nup133 | 1157 | 1-420 | Fugue | 1aoqA | 551 | 17-567 | 7 | 2.04 |  |  | 1.00 | -3.60 |  |
| Nup133 | 1157 | 560-1157 | mGTh | 1ldjA | 725 | 17-599 | 8.7 | 0.001 | -4.86 | -12.42 | 0.53 | -6.69 |  |
| Nup133 | 1157 | 609-1141 | mGTh | 1gw5A | 584 | 9-508 | 8 | 0.003 |  |  | 0.45 | -7.00 |  |
| Nup133 | 1157 | 585-1157 | mGTh | 1qgrA | 872 | 1-572 | 11 | 0.005 |  |  | 0.14 | -4.45 |  |
| Nup133 | 1157 | 631-1069 | mGTh | 1ho8 | 447 | 1-447 | 10 | 0.005 |  |  | 0.29 | -5.85 |  |
| Nup133 | 1157 | 520-1143 | mGTh | 1b3uA | 588 | 1-588 | 10 | 0.005 |  |  | 0.17 | -5.24 |  |
| Nup133 | 1157 | 650-1121 | mGTh | 1bk5A | 422 | 1-422 | 11 | 0.005 |  |  | 0.15 | -4.88 |  |
| Nup133 | 1157 | 421-1047 | mGTh | 1qbkB | 880 | 1-590 | 9 | 0.006 |  |  | 0.32 | -5.72 |  |
| Nup133 | 1157 | 810-1157 | mGTh | 1ib2A | 322 | 1-307 | 12 | 0.007 |  |  | 0.78 | -6.74 |  |
| Nup133 | 1157 | 601-928 | mGTh | 1fkmA | 322 | 249-630 | 15 | 0.008 |  |  | 0.77 | -5.81 |  |
| Nup133 | 1157 | 610-969 | mGTh | 1gw5A | 584 |  | 7.2 | 0.003 | -5.55 | -13.67 | 0.45 | -7.00 | -425.18 |
| Nup133 | 1157 | 525-975 | mGTh | 3bct | 457 | 1-457 | 7 | 0.009 |  |  | 0.14 | -5.94 |  |
| Nup133 | 1157 | 559-1157 | mGTh | 1ldjA | 725 | 17-599 | 8.7 | 0.001 | -4.86 | -12.42 | 0.53 | -6.69 |  |
| Nup133 | 1157 | 610-1141 | mGTh | 1gw5A | 584 | 9-508 | 7.2 | 0.003 | -5.55 | -13.67 | 0.45 | -7.00 | -651.85 |
| Nup133 | 1157 | 601-928 | mGTh | 1fkmA | 322 | 249-630 | 14.9 | 0.008 | -5.81 | -7.55 | 0.77 | -5.81 |  |
|  |  |  |  |  |  |  |  |  |  |  |  |  |  |
| Nup133 | 1157 | 610-1141 | Moulder0 | 1gw5A | 584 | 9-508 | 8 |  |  |  | 1.00 | -9.53 |  |
| Nup133 | 1157 | 610-1141 | Moulder1 | 1gw5A | 584 | 9-508 | 7 |  |  |  | 1.00 | -8.88 |  |
| Nup133 | 1157 | 610-1141 | Moulder2 | 1gw5A | 584 | 9-508 | 6 |  |  |  | 1.00 | -8.49 |  |
| Nup133 | 1157 | 610-1141 | Moulder3 | 1gw5A | 584 | 9-508 | 6 |  |  |  | 1.00 | -8.83 |  |
| Nup133 | 1157 | 610-1141 | Moulder4 | 1gw5A | 584 | 9-508 | 8 |  |  |  | 1.00 | -8.96 |  |
| Nup133 | 1157 | 1-300 | Moulder0 | 1erjA | 350 | 328-710 | 12 |  |  |  | 1.00 | -8.81 | -337.88 |
| Nup133 | 1157 | 1-300 | Moulder1 | 1erjA | 350 | 328-710 | 11 |  |  |  | 1.00 | -9.07 | -331.84 |
| Nup133 | 1157 | 1-300 | Moulder2 | 1erjA | 350 | 328-710 | 12 |  |  |  | 1.00 | -8.93 | -329.84 |
| Nup133 | 1157 | 1-300 | Moulder3 | 1erjA | 350 | 328-710 | 11 |  |  |  | 1.00 | -8.33 | -335.98 |
| Nup133 | 1157 | 1-300 | Moulder4 | 1erjA | 350 | 328-710 | 11 |  |  |  | 1.00 | -8.95 | -337.59 |
