# Supplementary material for: Components of Coated Vesicles and Nuclear Pore Complexes Share a Common Molecular Architecture
Source: PLoS Biol. 2004 Nov 2;2(12):e380. doi: 10.1371/journal.pbio.0020380 (PMC524472; doi:10.1371/journal.pbio.0020380)
Supplement: Table S3 — (115 KB DOC). [file pbio.0020380.st003.doc]

### Supplementary Table 3. Nup120 modeling results

The following annotations are used: mGTh, mGenThreader (McGuffin and Jones 2003); Fugue (Shi et al. 2001); Moulder# indicates the rank order of the MOULDER model (John and Sali 2003); SALIGN module of MODELLER (Marti-Renom et al. 2004); Prosa II Z-score (Sippl 1993), Dfire (Zhou and Zhou 2002); GA341 score (from 0 for models that tend to have an incorrect fold to 1 for models that tend to have at least the correct fold) and Melo Z-score (Melo et al. 2002).

| ***Nups*** | ***Prt size*** | ***Modeled***  ***fragment*** | ***Origin*** | ***Template*** | | | ***%id*** | ***Th Score*** | ***ProsaII Z-score*** | | ***GA341***  ***Score*** | ***Melo***  ***Z-score*** | ***Dfire*** |
| --- | --- | --- | --- | --- | --- | --- | --- | --- | --- | --- | --- | --- | --- |
|  |  |  |  | ***Id*** | ***Size*** | ***fragment*** |  |  | ***Model*** | ***Template*** |  |  |  |
| Nup120 | 1037 | 1-398 | mGTh | 1pguB | 608 | 18-388 | 7.8 | 0.076 | -3.6 | -7.2 | 0.03 | -3.44 |  |
| Nup120 | 1037 | 435-1033 | mGTh | 1b3uA | 588 | 1-565 | 8 | 0.0008 | -5.4 | -16.39 | 0.08 | -4.94 |  |
| Nup120 | 1037 | 1-398 | Salign | 1pguB | 608 | 18-388 | 7.8 | -5.4 | -4.58 | -7.2 | 0.25 | -5.19 |  |
| Nup120 | 1037 | 435-1033 | Salign | 1b3uA | 588 | 1-565 | 14.1 | -7.2 | -6.56 | -16.39 | 0.65 | -6.13 |  |
| Nup120 | 1037 | 481-1035 | mGTh | 1gw5B | 579 | 4-508 | 7 |  |  |  | 1.00 | -6.08 |  |
| Nup120 | 1037 | 531-1011 | mGTh | 1ee4A | 423 | 87-509 | 13 |  |  |  | 0.93 | -7.18 |  |
| Nup120 | 1037 | 531-1037 | mGTh | 1gw5A | 584 | 9-508 | 10 |  |  |  | 0.29 | -5.82 |  |
| Nup120 | 1037 | 48-420 | Fugue | 1k8kC | 354 | 3-353 | 6 | 9.15 |  |  | 0.04 | -5.59 |  |
| Nup120 | 1037 | 1-420 | Fugue | 1k32A | 1023 | 136-501 | 7 | 8.42 |  |  | 0.05 | -4.58 |  |
| Nup120 | 1037 | 2-403 | Fugue | 1gotB | 339 | 2-340 | 11 | 8.39 |  |  | 0.23 | -5.17 |  |
| Nup120 | 1037 | 1-407 | Fugue | 1nex | 444 | 295-744 | 10 | 6.42 |  |  | 0.01 | -1.91 |  |
| Nup120 | 1037 | 421-1037 | Fugue | 2btvA | 845 |  | 12 |  |  |  | 0.34 | -5.51 |  |
| Nup120 | 1037 | 421-1037 | Fugue | 1kko | 411 |  | 11 |  |  |  | 0.05 | -4.27 |  |
| Nup120 | 1037 | 421-1037 | Fugue | 1b3uA | 588 |  | 9 |  |  |  | 0.54 | -6.96 |  |
| Nup120 | 1037 | 421-1037 | Fugue | 7reqA | 725 | 4-556 | 11 |  |  |  | 0.48 | -6.22 |  |
| Nup120 | 1037 | 421-1037 | Fugue | 1h6kA | 728 | 43-788 | 11 |  |  |  | 0.57 | -6.32 |  |
| Nup120 | 1037 | 421-1037 | Fugue | 1jlaA | 457 | 3-553 | 15 |  |  |  | 0.04 | -2.49 |  |
| Nup120 | 1037 | 421-1037 | Fugue | 2btvB | 885 | 132-863 | 12 |  |  |  | 0.58 | -6.48 |  |
|  |  |  |  |  |  |  |  |  |  |  |  |  |  |
| Nup120 | 1037 | 481-1035 | Moulder0 | 1gw5B | 579 | 4-508 | 9 |  |  |  | 1.00 | -8.43 |  |
| Nup120 | 1037 | 481-1035 | Moulder1 | 1gw5B | 579 | 4-508 | 10 |  |  |  | 0.94 | -8.14 |  |
| Nup120 | 1037 | 481-1035 | Moulder2 | 1gw5B | 579 | 4-508 | 9 |  |  |  | 1.00 | -8.55 |  |
| Nup120 | 1037 | 481-1035 | Moulder3 | 1gw5B | 579 | 4-508 | 8 |  |  |  | 1.00 | -8.09 |  |
| Nup120 | 1037 | 481-1035 | Moulder4 | 1gw5B | 579 | 4-508 | 8 |  |  |  | 1.00 | -8.79 |  |
| Nup120 | 1037 | 531-1011 | Moulder0 | 1ee4A | 423 | 87-509 | 10 |  |  |  | 0.95 | -8.00 |  |
| Nup120 | 1037 | 531-1011 | Moulder1 | 1ee4A | 423 | 87-509 | 10 |  |  |  | 1.00 | -8.63 |  |
| Nup120 | 1037 | 531-1011 | Moulder2 | 1ee4A | 423 | 87-509 | 10 |  |  |  | 0.98 | -8.28 |  |
| Nup120 | 1037 | 531-1011 | Moulder3 | 1ee4A | 423 | 87-509 | 10 |  |  |  | 0.94 | -7.91 |  |
| Nup120 | 1037 | 531-1011 | Moulder4 | 1ee4A | 423 | 87-509 | 10 |  |  |  | 0.94 | -7.91 |  |
| Nup120 | 1037 | 1-398 | Moulder0 | 1pguB | 608 | 18-388 | 7 |  |  |  | 1.00 | -6.64 | -485.40 |
| Nup120 | 1037 | 1-398 | Moulder1 | 1pguB | 608 | 18-388 | 7 |  |  |  | 1.00 | -6.94 | -480.59 |
| Nup120 | 1037 | 1-398 | Moulder2 | 1pguB | 608 | 18-388 | 7 |  |  |  | 1.00 | -6.75 | -467.11 |
| Nup120 | 1037 | 1-398 | Moulder3 | 1pguB | 608 | 18-388 | 7 |  |  |  | 1.00 | -6.91 | -469.48 |
| Nup120 | 1037 | 1-398 | Moulder4 | 1pguB | 608 | 18-388 | 7 |  |  |  | 1.00 | -6.94 | -479.81 |
| Nup120 | 1037 | 435-1033 | Moulder0 | 1b3uA | 588 | 1-565 | 7 |  |  | -16.39 | 1.00 | -7.50 | -802.35 |
| Nup120 | 1037 | 435-1033 | Moulder1 | 1b3uA | 588 | 1-565 | 7 |  |  | -16.39 | 1.00 | -7.25 | -805.00 |
| Nup120 | 1037 | 435-1033 | Moulder2 | 1b3uA | 588 | 1-565 | 8 |  |  | -16.39 | 1.00 | -7.19 | -823.80 |
| Nup120 | 1037 | 435-1033 | Moulder3 | 1b3uA | 588 | 1-565 | 7 |  |  | -16.39 | 1.00 | -6.43 | -799.24 |
| Nup120 | 1037 | 435-1033 | Moulder4 | 1b3uA | 588 | 1-565 | 8 |  |  | -16.39 | 1.00 | -6.85 | -816.26 |
