# Supplementary material for: Components of Coated Vesicles and Nuclear Pore Complexes Share a Common Molecular Architecture
Source: PLoS Biol. 2004 Nov 2;2(12):e380. doi: 10.1371/journal.pbio.0020380 (PMC524472; doi:10.1371/journal.pbio.0020380)
Supplement: Table S4 — (132 KB DOC). [file pbio.0020380.st004.doc]

### Supplementary Table 4. Nup85 modeling results

| ***Nups*** | ***Prt size*** | ***Modeled***  ***fragment*** | ***Origin*** | ***Template*** | | | ***%id*** | ***Th Score*** | ***ProsaII Z-score*** | | ***GA341***  ***Score*** | ***Melo***  ***Z-score*** | ***Dfire*** |
| --- | --- | --- | --- | --- | --- | --- | --- | --- | --- | --- | --- | --- | --- |
|  |  |  |  | ***Id*** | ***Size*** | ***fragment*** |  |  | ***Model*** | ***Template*** |  |  |  |
| Nup85 | 744 | 1-131 | mGTh | 1e0cA | 144 | 1-117 | 13.7 | 0.34 | -2 | -9.32 | 0.06 | -2.26 | -126.68 |
| Nup85 | 744 | 1-131 | mGTh | 1ez1A | 389 | 190-320 | 11.5 | 0.451 | -1 | -6.56 | 0.01 | -0.91 |  |
| Nup85 | 744 | 203-744 | mGTh | 1gw5A | 584 | 9-508 | 8.7 | 0.0004 | -6 | -13.67 | 0.98 | -8.24 | -637.44 |
| Nup85 | 744 | 1-131 | Salign | 1e0cA | 144 | 1-117 | 8.8 | -1.4 | -2.14 | -9.32 | 0.02 | -2.23 | -126.68 |
| Nup85 | 744 | 203-744 | Salign | 1gw5A | 584 | 9-508 | 7.3 | -1.6 | -4.08 | -13.67 | 0.01 | -3.43 |  |
| Nup85 | 744 | 1-152 | Salign | 1gw5M | 142 | 1-142 | 13 |  | -2.8 | -6.73 | 0.02 | -1.82 | -144.09 |
| Nup85 | 744 | 1-152 | Salign | 1gw5S | 142 | 1-142 | 19 |  | -1.25 | -5.28 | 0.02 | -0.85 | -153.74 |
| Nup85 | 744 | 1-152 | Salign | 1gw5S | 142 | 1-142 | 13 |  | -1.25 | -5.28 | 0.04 | -2.57 | -153.74 |
| Nup85 | 744 | 1-152 | Moulder0 | 1gw5S | 142 | 1-142 | 13 |  | -4.92 | -5.28 | 0.28 | -5.48 | -150.19 |
| Nup85 | 744 | 1-152 | Moulder1 | 1gw5S | 142 | 1-142 | 13 |  | -4.71 | -5.28 | 0.53 | -5.57 | -149.81 |
| Nup85 | 744 | 1-152 | Moulder2 | 1gw5S | 142 | 1-142 | 13 |  | -4.33 | -5.28 | 0.30 | -5.74 | -150.21 |
| Nup85 | 744 | 1-152 | Moulder3 | 1gw5S | 142 | 1-142 | 13 |  | -4.37 | -5.28 | 0.58 | -5.63 | -154.49 |
| Nup85 | 744 | 1-152 | Moulder4 | 1gw5S | 142 | 1-142 | 13 |  | -4 | -5.28 | 0.34 | -5.53 | -160.95 |
| Nup85 | 744 | 1-150 | Fugue | 1fgs | 128 | 297-425 | 11 | 2.21 | -3.48 |  | 0.11 | -3.64 | -155.34 |
| Nup85 | 744 | 1-150 | Fugue | 1f5sA | 209 | 2-211 | 11 | 2.12 | -3.47 |  | 0.12 | -3.78 | -140.34 |
| Nup85 | 744 | 1-150 | Fugue | 1gmxA | 108 | 1-108 | 15 | 2.46 | -3.88 |  | 0.12 | -3.72 | -143.19 |
| Nup85 | 744 | 60-130 | Fugue | 1nyhA | 76 | 1271-1346 | 14 | 2.45 | 1.41 |  | 0.00 | -0.21 | -78.85 |
| Nup85 | 744 | 1-150 | Fugue | 1ddt | 154 | 381-535 | 17 | 2.76 | -3.31 |  | 0.24 | -3.52 | -150.81 |
| Nup85 | 744 | 1-150 | Fugue | 1a34A | 147 | 13-159 | 17 | 2.41 | -2.92 |  | 0.07 | -3.08 | -125.23 |
| Nup85 | 744 | 1-150 | Fugue | 1b24A | 91 | 7-99 | 16 | 2.15 | -1.19 |  | 0.01 | -1.15 | -104.16 |
| Nup85 | 744 | 1-150 | Fugue | 1hcd | 118 | 1-118 | 17 | 2.25 | -2.41 |  | 0.23 | -3.32 | -138.22 |
| Nup85 | 744 | 1-150 | Fugue | 1kafA | 108 | 104-211 | 20 | 2.34 | -3.88 |  | 0.23 | -3.72 | -143.19 |
| Nup85 | 744 | 1-150 | Fugue | 1pda | 120 | 2-219 | 13 | 3.2 | -2.99 |  | 0.21 | -4.11 | -128.71 |
|  |  |  |  |  |  |  |  |  |  |  |  |  |  |
| Nup85 | 744 | 203-744 | mGTh | 1gw5A | 584 | 9-508 | 8.7 | 0.0004 | -6 | -13.67 | 0.98 | -8.24 | -637.44 |
| Nup85 | 744 | 203-744 | Moulder0 | 1gw5A | 584 | 9-508 | 10 |  |  | -13.67 | 1.00 | -11.22 | -673.82 |
| Nup85 | 744 | 203-744 | Moulder1 | 1gw5A | 584 | 9-508 | 10 |  |  | -13.67 | 1.00 | -11.33 | -675.65 |
| Nup85 | 744 | 203-744 | Moulder2 | 1gw5A | 584 | 9-508 | 10 |  | -7.39 | -13.67 | 1.00 | -11.84 | -680.14 |
| Nup85 | 744 | 203-744 | Moulder3 | 1gw5A | 584 | 9-508 | 9 |  |  | -13.67 | 1.00 | -11.75 | -673.40 |
| Nup85 | 744 | 203-744 | Moulder4 | 1gw5A | 584 | 9-508 | 10 |  |  | -13.67 | 1.00 | -11.55 | -653.06 |
|  |  |  |  |  |  |  |  |  |  |  |  |  |  |
| Nup85 | 744 | 1-142 | Salign | 1dcq | 276 | 247-394 | 3 |  |  |  | 0.00 | 0.64 | -136.29 |
| Nup85 | 744 | 189-301 | Salign | 1nwm | 105 | 191-h301 | 7 |  |  |  |  | -3.06 |  |
| Nup85 | 744 | 203-315 | Moulder0 | 1gw5A | 584 | 9-508 | 10 |  |  |  |  | -4.58 |  |
|  |  |  |  |  |  |  |  |  |  |  |  |  |  |
| Nup85 | 744 | 100-744 | Fugue | 1b3uA | 588 | 1-583 | 8 | 4.19 |  |  | 1.00 | -8.33 |  |
| Nup85 | 744 | 99-744 | Fugue | 1qgrA | 871 | 149-838 | 8 | 3.39 |  |  | 1.00 | -7.30 |  |
| Nup85 | 744 | 101-736 | Fugue | 1i7wC | 509 | 143-663 | 10 | 3.37 |  |  | 0.84 | -7.81 |  |
| Nup85 | 744 | 116-736 | Fugue | 1jdhA | 508 | 135-663 | 9 | 3.18 |  |  | 1.00 | -5.19 |  |
|  |  |  |  |  |  |  |  |  |  |  |  |  |  |
| Nup85 | 744 | 100-744 | Fugue | 1b3uA | 588 | 1-583 | 8 | 4.19 |  |  | 1.00 | -8.33 |  |
| Nup85 | 744 | 100-744 | Moulder0 | 1b3uA | 588 | 1-583 | 8 |  |  |  | 1.00 | -7.45 |  |
| Nup85 | 744 | 100-744 | Moulder1 | 1b3uA | 588 | 1-583 | 9 |  |  |  | 1.00 | -7.26 |  |
| Nup85 | 744 | 100-744 | Moulder2 | 1b3uA | 588 | 1-583 | 8 |  |  |  | 1.00 | -7.46 |  |
| Nup85 | 744 | 100-744 | Moulder3 | 1b3uA | 588 | 1-583 | 8 |  |  |  | 1.00 | -7.62 |  |
| Nup85 | 744 | 100-744 | Moulder4 | 1b3uA | 588 | 1-583 | 9 |  |  |  | 1.00 | -7.00 |  |

In the following tables, the following annotations are used: mGTh, mGenThreader (McGuffin and Jones 2003); Fugue (Shi et al. 2001); Moulder# indicates the rank order of the MOULDER model (John and Sali 2003); SALIGN module of MODELLER (Marti-Renom et al. 2004); Prosa II Z-score (Sippl 1993), Dfire (Zhou and Zhou 2002); GA341 score (from 0 for models that tend to have an incorrect fold to 1 for models that tend to have at least the correct fold) and Melo Z-score (Melo et al. 2002).
