# Supplementary material for: Components of Coated Vesicles and Nuclear Pore Complexes Share a Common Molecular Architecture
Source: PLoS Biol. 2004 Nov 2;2(12):e380. doi: 10.1371/journal.pbio.0020380 (PMC524472; doi:10.1371/journal.pbio.0020380)
Supplement: Table S5 — (124 KB DOC). [file pbio.0020380.st005.doc]

### Supplementary Table 5. Nup84 modeling results

In the following tables, the following annotations are used: mGTh, mGenThreader (McGuffin and Jones 2003); Fugue (Shi et al. 2001); Moulder# indicates the rank order of the MOULDER model (John and Sali 2003); SALIGN module of MODELLER (Marti-Renom et al. 2004); Prosa II Z-score (Sippl 1993), Dfire (Zhou and Zhou 2002); GA341 score (from 0 for models that tend to have an incorrect fold to 1 for models that tend to have at least the correct fold) and Melo Z-score (Melo et al. 2002).

| ***Nups*** | ***Prt size*** | ***Modeled***  ***fragment*** | ***Origin*** | ***Template*** | | | ***%id*** | ***Th Score*** | ***ProsaII Z-score*** | | ***GA341***  ***Score*** | ***Melo***  ***Z-score*** | ***Dfire*** |
| --- | --- | --- | --- | --- | --- | --- | --- | --- | --- | --- | --- | --- | --- |
|  |  |  |  | ***Id*** | ***Size*** | ***fragment*** |  |  | ***Model*** | ***Template*** |  |  |  |
| Nup84 | 726 | 53-695 | mGTh | 1b3uA | 588 | 1-588 | 11.2 | 0.0004 | -7.35 | -16.39 | 0.50 | -6.40 |  |
| Nup84 | 726 | 236-726 | mGTh | 1gw5A | 584 | 9-490 | 8 | 0.0008 |  |  | 1.00 | -6.31 |  |
| Nup84 | 726 | 322-715 | mGTh | 1ee4A | 423 | 87-509 | 11.1 | 0.0009 |  |  | 0.95 | -7.85 |  |
|  |  |  |  |  |  |  |  |  |  |  |  |  |  |
| Nup84 | 726 | 1-726 | Fugue | 1qgrA | 871 | 95-853 |  | 2.45 |  |  |  |  |  |
| Nup84 | 726 | 53-695 | Salign | 1b3uA | 588 | 1-588 | 7.5 | -0.4 | -1.72 | -16.39 | 0.00 | -0.81 |  |
|  |  |  |  |  |  |  |  |  |  |  |  |  |  |
| Nup84 | 726 | 1-726 | Fugue | 1g8x | 1009 | 2-1010 | 11 | 2.53 |  |  | 0.77 | -7.49 | -838.74 |
| Nup84 | 726 | 9-726 | Fugue | 1i7w | 509 | 149-662 | 8 | 2.03 |  |  | 0.04 | -4.96 | -3.49 |
| Nup84 | 726 | 1-726 | Fugue | 1jdhA | 508 | 135-663 | 8 | 2.18 |  |  | 0.08 | -5.64 | -89.10 |
| Nup84 | 726 | 1-726 | Fugue | 1n1b | 534 | 64-598 | 8 | 1.98 |  |  | 0.01 | -2.93 | 16.39 |
| Nup84 | 726 | 1-726 | Fugue | 1qgrA | 871 | 1-876 | 10 | 2.45 |  |  | 0.19 | -5.29 | -926.29 |
| Nup84 | 726 | 1-726 | Fugue | 5eau | 523 | 21-548 | 8 | 1.98 |  |  | 0.00 | -2.65 | 70.99 |
|  |  |  |  |  |  |  |  |  |  |  |  |  |  |
| Nup84 | 726 | 137-726 | Fugue | 1csh | 435 | 3-437 | 8 | 2.74 |  |  |  | -3.62 |  |
| Nup84 | 726 | 137-726 | Fugue | 1aj8 | 371 | 6-376 | 12 | 2.74 |  |  | 0.02 | -2.84 |  |
| Nup84 | 726 | 137-726 | Fugue | 1a59 | 377 | 2-378 | 10 | 2.74 |  |  | 0.01 | -2.54 |  |
| Nup84 | 726 | 137-726 | Fugue | 1qgrA | 872 | 1-876 | 10 | 2.67 |  |  | 0.06 | -3.72 |  |
| Nup84 | 726 | 137-726 | Fugue | 1b3uA | 588 | 1-588 | 9 | 2.49 |  |  | 0.85 | -7.94 |  |
| Nup84 | 726 | 137-726 | Fugue | 1qbk | 880 | 3-889 | 9 | 2.23 |  |  | 0.01 | -2.54 |  |
| Nup84 | 726 | 137-726 | Fugue | 1b89 | 321 | 1182-1516 | 10 | 2.06 |  |  | 0.03 | -4.19 |  |
|  |  |  |  |  |  |  |  |  |  |  |  |  |  |
| Nup84 | 726 | 137-726 | Moulder0 | 1b3uA | 588 | 1-588 | 10 |  |  |  | 0.86 | -8.04 |  |
| Nup84 | 726 | 137-726 | Moulder1 | 1b3uA | 588 | 1-588 | 10 |  |  |  | 0.83 | -7.93 |  |
| Nup84 | 726 | 137-726 | Moulder2 | 1b3uA | 588 | 1-588 | 9 |  |  |  | 1.00 | -7.93 |  |
| Nup84 | 726 | 137-726 | Moulder3 | 1b3uA | 588 | 1-588 | 9 |  |  |  | 1.00 | -8.07 |  |
| Nup84 | 726 | 137-726 | Moulder4 | 1b3uA | 588 | 1-588 | 9 |  |  |  | 1.00 | -8.08 |  |
|  |  |  |  |  |  |  |  |  |  |  |  |  |  |
| Nup84 | 727 | 237-726 | Moulder0 | 1gw5A | 585 | 9-490 | 9 |  |  |  | 1.00 | -8.78 |  |
| Nup84 | 728 | 238-726 | Moulder1 | 1gw5A | 586 | 9-490 | 9 |  |  |  | 1.00 | -8.47 |  |
| Nup84 | 729 | 239-726 | Moulder2 | 1gw5A | 587 | 9-490 | 10 |  |  |  | 1.00 | -8.73 |  |
| Nup84 | 730 | 240-726 | Moulder3 | 1gw5A | 588 | 9-490 | 10 |  |  |  | 0.97 | -8.23 |  |
| Nup84 | 731 | 241-726 | Moulder4 | 1gw5A | 589 | 9-490 | 9 |  |  |  | 1.00 | -9.14 |  |
|  |  |  |  |  |  |  |  |  |  |  |  |  |  |
| Nup84 | 726 | 322-715 | Moulder0 | 1ee4A | 423 | 87-509 | 11 |  |  |  | 1.00 | -10.29 |  |
| Nup84 | 726 | 322-715 | Moulder1 | 1ee4A | 423 | 87-509 | 11 |  |  |  | 1.00 | -10.15 |  |
| Nup84 | 726 | 322-715 | Moulder2 | 1ee4A | 423 | 87-509 | 12 |  |  |  | 1.00 | -9.63 |  |
| Nup84 | 726 | 322-715 | Moulder3 | 1ee4A | 423 | 87-509 | 13 |  |  |  | 1.00 | -9.85 |  |
| Nup84 | 726 | 322-715 | Moulder4 | 1ee4A | 423 | 87-509 | 10 |  |  |  | 1.00 | -10.92 |  |
|  |  |  |  |  |  |  |  |  |  |  |  |  |  |
| Nup84 | 726 | 53-695 | Moulder0 | 1b3uA | 588 | 1-588 | 10 |  |  |  | 0.86 | -8.04 | -773.84 |
| Nup84 | 726 | 53-695 | Moulder1 | 1b3uA | 588 | 1-588 | 10 |  |  |  | 0.83 | -7.93 | -786.62 |
| Nup84 | 726 | 53-695 | Moulder2 | 1b3uA | 588 | 1-588 | 9 |  |  |  | 1.00 | -7.93 | -773.08 |
| Nup84 | 726 | 53-695 | Moulder3 | 1b3uA | 588 | 1-588 | 9 |  |  |  | 1.00 | -8.07 | -795.09 |
| Nup84 | 726 | 53-695 | Moulder4 | 1b3uA | 588 | 1-588 | 9 |  |  |  | 1.00 | -8.08 | -770.25 |
