# Supplementary material for: Components of Coated Vesicles and Nuclear Pore Complexes Share a Common Molecular Architecture
Source: PLoS Biol. 2004 Nov 2;2(12):e380. doi: 10.1371/journal.pbio.0020380 (PMC524472; doi:10.1371/journal.pbio.0020380)
Supplement: Table S6 — (93 KB DOC). [file pbio.0020380.st006.doc]

### Supplementary Table 6. Nup145C modeling results

In the following tables, the following annotations are used: mGTh, mGenThreader (McGuffin and Jones 2003); Fugue (Shi et al. 2001); Moulder# indicates the rank order of the MOULDER model (John and Sali 2003); SALIGN module of MODELLER (Marti-Renom et al. 2004); Prosa II Z-score (Sippl 1993), Dfire (Zhou and Zhou 2002); GA341 score (from 0 for models that tend to have an incorrect fold to 1 for models that tend to have at least the correct fold) and Melo Z-score (Melo et al. 2002)

| ***Nups*** | ***Prt size*** | ***Modeled***  ***fragment*** | ***Origin*** | ***Template*** | | | ***%id*** | ***Th Score*** | ***ProsaII Z-score*** | | ***GA341***  ***Score*** | ***Melo***  ***Z-score*** | ***Dfire*** |
| --- | --- | --- | --- | --- | --- | --- | --- | --- | --- | --- | --- | --- | --- |
|  |  |  |  | ***Id*** | ***Size*** | ***fragment*** |  |  | ***Model*** | ***Template*** |  |  |  |
| Nup145C | 712 | 1-354 | mGTh | 1qgrA | 872 | 94-445 | 9.9 | 0.028 | -5.2 | -12.3 | 0.14 | -4.79 |  |
| Nup145C | 712 | 1-712 | mGTh | 1qbkB | 880 | 124-864 | 9.8 | 0.0006 | -5.88 | -1.19 | 0.41 | -5.89 |  |
| Nup145C | 712 | 234-690 | mGTh | 1bk5A | 422 | 89-509 | 13.5 | 0.0009 | -6.5 | -14.83 | 1.00 | -8.19 | -592.57 |
| Nup145C | 712 | 215-712 | mGTh | 1gw5A | 584 | 9-508 | 9 | 0.002 |  |  | 1.00 | -5.73 |  |
| Nup145C | 712 | 205-712 | mGTh | 1gw5B | 579 | 4-508 | 8 | 0.002 |  |  | 1.00 | -7.38 |  |
| Nup145C | 712 | 255-712 | Fugue | 1b3uA | 588 | 1-588 | 8 | 2.81 |  |  | 0.73 | -7.75 |  |
| Nup145C | 712 | 255-712 | Fugue | 1ct9A | 497 | 1-516 | 8 | 2.1 |  |  | 0.04 | -3.44 |  |
| Nup145C | 712 | 255-712 | Fugue | 1dl5A | 317 | 1-317 | 7 | 2.12 |  |  | 0.08 | -4.74 |  |
| Nup145C | 712 | 255-712 | Fugue | 1gw5A | 500 | 9-508 | 7 | 2.21 |  |  | 0.18 | -6.25 |  |
|  |  |  |  |  |  |  |  |  |  |  |  |  |  |
| Nup145C | 712 | 1-354 | Salign | 1qgrA | 872 | 94-445 | 6.6 | -2.1 | -0.3 | -12.3 | 0.00 | -1.30 |  |
| Nup145C | 712 | 1-712 | Salign | 1qbkB | 880 | 124-864 | 8.8 | -4.6 | -3.79 | -1.19 | 0.03 | -3.32 |  |
| Nup96 | 937 | 390-937 | mGTh | 1gw5A | 584 | 1-496 | 8.6 | 0.001 | -5.08 | -13.6 | 0.22 | -5.39 |  |
| Nup145C | 712 | 215-712 | Moulder0 | 1gw5A | 584 | 9-508 | 11 |  |  |  | 1.00 | -8.91 |  |
| Nup145C | 712 | 215-712 | Moulder1 | 1gw5A | 584 | 9-508 | 11 |  |  |  | 1.00 | -9.51 |  |
| Nup145C | 712 | 215-712 | Moulder2 | 1gw5A | 584 | 9-508 | 11 |  |  |  | 1.00 | -8.67 |  |
| Nup145C | 712 | 215-712 | Moulder3 | 1gw5A | 584 | 9-508 | 11 |  |  |  | 1.00 | -9.64 |  |
| Nup145C | 712 | 215-712 | Moulder4 | 1gw5A | 584 | 9-508 | 12 |  |  |  | 1.00 | -9.05 |  |
| Nup145C | 712 | 1-712 | Moulder0 | 1qbkB | 880 | 124-864 | 9 |  |  |  | 1.00 | -10.47 |  |
| Nup145C | 712 | 1-712 | Moulder1 | 1qbkB | 880 | 124-864 | 10 |  |  |  | 1.00 | -10.73 |  |
| Nup145C | 712 | 1-712 | Moulder2 | 1qbkB | 880 | 124-864 | 10 |  |  |  | 1.00 | -10.51 |  |
| Nup145C | 712 | 1-712 | Moulder3 | 1qbkB | 880 | 124-864 | 10 |  |  |  | 1.00 | -10.38 |  |
| Nup145C | 712 | 1-712 | Moulder4 | 1qbkB | 880 | 124-864 | 9 |  |  |  | 1.00 | -10.33 |  |
| Nup145C | 712 | 234-690 | Moulder0 | 1bk5A | 422 | 89-509 | 11 |  |  |  | 1.00 | -10.07 | -607.58 |
| Nup145C | 712 | 234-690 | Moulder1 | 1bk5A | 422 | 89-509 | 12 |  |  |  | 1.00 | -8.54 | -579.92 |
| Nup145C | 712 | 234-690 | Moulder2 | 1bk5A | 422 | 89-509 | 13 |  |  |  | 1.00 | -10.38 | -601.16 |
| Nup145C | 712 | 234-690 | Moulder3 | 1bk5A | 422 | 89-509 | 13 |  |  |  | 1.00 | -8.83 | -593.94 |
| Nup145C | 712 | 234-690 | Moulder4 | 1bk5A | 422 | 89-509 | 12 |  |  |  | 1.00 | -9.63 | -601.25 |
