# Supplementary material for: Components of Coated Vesicles and Nuclear Pore Complexes Share a Common Molecular Architecture
Source: PLoS Biol. 2004 Nov 2;2(12):e380. doi: 10.1371/journal.pbio.0020380 (PMC524472; doi:10.1371/journal.pbio.0020380)
Supplement: Table S7 — (144 KB DOC). [file pbio.0020380.st007.doc]

### Supplementary Table 7. Human and plant Nup84 complex proteins modeling results

The following annotations are used: mGTh, mGenThreader (McGuffin and Jones 2003); Fugue (Shi et al. 2001); Moulder# indicates the rank order of the MOULDER model (John and Sali 2003); SALIGN module of MODELLER (Marti-Renom et al. 2004); Prosa II Z-score (Sippl 1993), Dfire (Zhou and Zhou 2002); GA341 score (from 0 for models that tend to have an incorrect fold to 1 for models that tend to have at least the correct fold) and Melo Z-score (Melo et al. 2002).

| ***Nups*** | ***SwissProt***  ***identifier*** | ***Prt size*** | ***Modeled***  ***fragment*** | ***Origin*** | ***Template*** | | | ***%id*** | ***GA341***  ***Score*** | ***Melo***  ***Z-score*** |
| --- | --- | --- | --- | --- | --- | --- | --- | --- | --- | --- |
|  |  |  |  |  | ***Id*** | ***Size*** | ***fragment*** |  |  |  |
| N133_human | Q8WUM0 | 1156 | 1-302 | Moulder0 | 1erjA | 350 | 328-710 | 8 | 1.00 | -5.85 |
| N133_human | Q8WUM0 | 1156 | 1-302 | Moulder1 | 1erjA | 350 | 328-710 | 8 | 1.00 | -6.15 |
| N133_human | Q8WUM0 | 1156 | 1-302 | Moulder2 | 1erjA | 350 | 328-710 | 6 | 1.00 | -6.10 |
| N133_human | Q8WUM0 | 1156 | 1-302 | Moulder3 | 1erjA | 350 | 328-710 | 7 | 1.00 | -6.44 |
| N133_human | Q8WUM0 | 1156 | 1-302 | Moulder4 | 1erjA | 350 | 328-710 | 8 | 1.00 | -6.52 |
|  |  |  |  |  |  |  |  |  |  |  |
| N133_human | Q8WUM0 | 1156 | 670-1150 | Moulder0 | 1gw5A | 584 | 11-508 | 7 | 1.00 | -7.79 |
| N133_human | Q8WUM0 | 1156 | 670-1150 | Moulder1 | 1gw5A | 584 | 11-508 | 7 | 1.00 | -7.33 |
| N133_human | Q8WUM0 | 1156 | 670-1150 | Moulder2 | 1gw5A | 584 | 11-508 | 7 | 1.00 | -7.08 |
| N133_human | Q8WUM0 | 1156 | 670-1150 | Moulder3 | 1gw5A | 584 | 11-508 | 7 | 1.00 | -7.20 |
| N133_human | Q8WUM0 | 1156 | 670-1150 | Moulder4 | 1gw5A | 584 | 11-508 | 8 | 1.00 | -7.58 |
|  |  |  |  |  |  |  |  |  |  |  |
| N160_human | Q12769 | 1316 | 1-414 | Moulder0 | 1pguB | 608 | 18-388 | 5 | 1.00 | -6.06 |
| N160_human | Q12769 | 1316 | 1-414 | Moulder1 | 1pguB | 608 | 18-388 | 5 | 1.00 | -5.57 |
| N160_human | Q12769 | 1316 | 1-414 | Moulder2 | 1pguB | 608 | 18-388 | 6 | 1.00 | -6.00 |
| N160_human | Q12769 | 1316 | 1-414 | Moulder3 | 1pguB | 608 | 18-388 | 4 | 1.00 | -5.43 |
| N160_human | Q12769 | 1316 | 1-414 | Moulder4 | 1pguB | 608 | 18-388 | 4 | 1.00 | -5.65 |
|  |  |  |  |  |  |  |  |  |  |  |
| N160_human | Q12769 | 1316 | 515-1156 | Moulder0 | 1gw5B | 579 | 4-504 | 8 | 1.00 | -7.05 |
| N160_human | Q12769 | 1316 | 515-1156 | Moulder1 | 1gw5B | 579 | 4-504 | 6 | 1.00 | -7.11 |
| N160_human | Q12769 | 1316 | 515-1156 | Moulder2 | 1gw5B | 579 | 4-504 | 6 | 1.00 | -7.13 |
| N160_human | Q12769 | 1316 | 515-1156 | Moulder3 | 1gw5B | 579 | 4-504 | 6 | 1.00 | -7.21 |
| N160_human | Q12769 | 1316 | 515-1156 | Moulder4 | 1gw5B | 579 | 4-504 | 7 | 1.00 | -6.81 |
|  |  |  |  |  |  |  |  |  |  |  |
| N133_plant | Q9SJ43 | 1234 | 735-1224 | Moulder0 | 1gw5A | 584 | 11-508 | 8 | 1.00 | -7.54 |
| N133_plant | Q9SJ43 | 1234 | 735-1224 | Moulder1 | 1gw5A | 584 | 11-508 | 8 | 1.00 | -7.89 |
| N133_plant | Q9SJ43 | 1234 | 735-1224 | Moulder2 | 1gw5A | 584 | 11-508 | 8 | 1.00 | -7.40 |
| N133_plant | Q9SJ43 | 1234 | 735-1224 | Moulder3 | 1gw5A | 584 | 11-508 | 7 | 1.00 | -6.67 |
| N133_plant | Q9SJ43 | 1234 | 735-1224 | Moulder4 | 1gw5A | 584 | 11-508 | 8 | 1.00 | -7.07 |
|  |  |  |  |  |  |  |  |  |  |  |
| N133_plant | Q9SJ43 | 1234 | 1-312 | Moulder0 | 1erjA | 350 | 328-710 | 8 | 1.00 | 4.79 |
| N133_plant | Q9SJ43 | 1234 | 1-312 | Moulder1 | 1erjA | 350 | 328-710 | 8 | 1.00 | -4.44 |
| N133_plant | Q9SJ43 | 1234 | 1-312 | Moulder2 | 1erjA | 350 | 328-710 | 8 | 1.00 | -4.54 |
| N133_plant | Q9SJ43 | 1234 | 1-312 | Moulder3 | 1erjA | 350 | 328-710 | 8 | 1.00 | -4.51 |
| N133_plant | Q9SJ43 | 1234 | 1-312 | Moulder4 | 1erjA | 350 | 328-710 | 8 | 1.00 | -4.73 |
|  |  |  |  |  |  |  |  |  |  |  |
| N75_human | Q9BW27 | 656 | 161-656 | Moulder0 | 1gw5A | 584 | 9-508 | 6 | 1.00 | -5.22 |
| N75_human | Q9BW27 | 656 | 161-656 | Moulder1 | 1gw5A | 584 | 9-508 | 6 | 1.00 | -5.71 |
| N75_human | Q9BW27 | 656 | 161-656 | Moulder2 | 1gw5A | 584 | 9-508 | 6 | 1.00 | -4.05 |
| N75_human | Q9BW27 | 656 | 161-656 | Moulder3 | 1gw5A | 584 | 9-508 | 6 | 1.00 | -5.63 |
| N75_human | Q9BW27 | 656 | 161-656 | Moulder4 | 1gw5A | 584 | 9-508 | 6 | 1.00 | -4.22 |
|  |  |  |  |  |  |  |  |  |  |  |
| N85_thaliana | Q8RXH2 | 716 | 188-716 | Moulder0 | 1gw5A | 584 | 9-508 | 6 | 1.00 | -5.67 |
| N85_thaliana | Q8RXH2 | 716 | 188-716 | Moulder1 | 1gw5A | 584 | 9-508 | 6 | 1.00 | -5.50 |
| N85_thaliana | Q8RXH2 | 716 | 188-716 | Moulder2 | 1gw5A | 584 | 9-508 | 6 | 1.00 | -5.32 |
| N85_thaliana | Q8RXH2 | 716 | 188-716 | Moulder3 | 1gw5A | 584 | 9-508 | 6 | 1.00 | -6.00 |
| N85_thaliana | Q8RXH2 | 716 | 188-716 | Moulder4 | 1gw5A | 584 | 9-508 | 6 | 1.00 | -5.30 |
|  |  |  |  |  |  |  |  |  |  |  |
| N84_thaliana | Q8L748 | 1101 | 385-1093 | Moulder0 | 1gw5A | 584 | 9-490 | 7 | 1.00 | -7.33 |
| N84_thaliana | Q8L748 | 1101 | 385-1093 | Moulder1 | 1gw5A | 585 | 9-490 | 8 | 1.00 | -7.68 |
| N84_thaliana | Q8L748 | 1101 | 385-1093 | Moulder2 | 1gw5A | 586 | 9-490 | 8 | 1.00 | -6.14 |
| N84_thaliana | Q8L748 | 1101 | 385-1093 | Moulder3 | 1gw5A | 587 | 9-490 | 7 | 1.00 | -5.73 |
| N84_thaliana | Q8L748 | 1101 | 385-1093 | Moulder4 | 1gw5A | 588 | 9-490 | 7 | 1.00 | -8.01 |
|  |  |  |  |  |  |  |  |  |  |  |
| N107_human | P57740 | 925 | 400-913 | Moulder0 | 1gw5A | 584 | 9-490 | 8 | 1.00 | -6.31 |
| N107_human | P57740 | 925 | 400-913 | Moulder1 | 1gw5A | 585 | 9-490 | 7 | 1.00 | -7.39 |
| N107_human | P57740 | 925 | 400-913 | Moulder2 | 1gw5A | 586 | 9-490 | 6 | 1.00 | -7.59 |
| N107_human | P57740 | 925 | 400-913 | Moulder3 | 1gw5A | 587 | 9-490 | 7 | 1.00 | -6.76 |
| N107_human | P57740 | 925 | 400-913 | Moulder4 | 1gw5A | 588 | 9-490 | 7 | 1.00 | -6.88 |
|  |  |  |  |  |  |  |  |  |  |  |
| N96_human | P52948 | 937 | 392-909 | Moulder0 | 1bk5A | 422 | 89-509 | 6 | 1.00 | -8.53 |
| N96_human | P52948 | 937 | 392-909 | Moulder1 | 1bk5A | 422 | 89-509 | 6 | 1.00 | -8.20 |
| N96_human | P52948 | 937 | 392-909 | Moulder2 | 1bk5A | 422 | 89-509 | 7 | 1.00 | -8.48 |
| N96_human | P52948 | 937 | 392-909 | Moulder3 | 1bk5A | 422 | 89-509 | 6 | 1.00 | -7.81 |
| N96_human | P52948 | 937 | 392-909 | Moulder4 | 1bk5A | 422 | 89-509 | 7 | 1.00 | -8.78 |
|  |  |  |  |  |  |  |  |  |  |  |
| N145_thalian | Q8LLD0 | 859 | 314-828 | Moulder0 | 1bk5A | 422 | 89-509 | 9 | 1.00 | -6.35 |
| N145_thalian | Q8LLD0 | 859 | 314-828 | Moulder1 | 1bk5A | 422 | 89-509 | 9 | 1.00 | -6.31 |
| N145_thalian | Q8LLD0 | 859 | 314-828 | Moulder2 | 1bk5A | 422 | 89-509 | 8 | 1.00 | -5.94 |
| N145_thalian | Q8LLD0 | 859 | 314-828 | Moulder3 | 1bk5A | 422 | 89-509 | 8 | 1.00 | -6.51 |
| N145_thalian | Q8LLD0 | 859 | 314-828 | Moulder4 | 1bk5A | 422 | 89-509 | 8 | 1.00 | -6.24 |
